# An Integrated Multi-OMICS Approach Highlights Elevated Non-Esterified Fatty Acids Impact BeWo Trophoblast Metabolism and Lipid Processing

**DOI:** 10.3390/metabo13080883

**Published:** 2023-07-25

**Authors:** Zachary J. W. Easton, Ousseynou Sarr, Lin Zhao, Adriana Zardini Buzatto, Xian Luo, Shuang Zhao, Liang Li, Timothy R. H. Regnault

**Affiliations:** 1 Department of Physiology and Pharmacology, Western University, Medical Sciences Building Room 216, London, ON N6A 5C1, Canada; zeaston@uwo.ca (Z.J.W.E.); osarr@uwo.ca (O.S.); lzhao3@uwo.ca (L.Z.); 2 The Metabolomics Innovation Centre (TMIC), University of Alberta, Edmonton, AB T6G 2G2, Canada; zardinib@ualberta.ca (A.Z.B.); xluo2@ualberta.ca (X.L.); szhao1@ualberta.ca (S.Z.); liang.li@ualberta.ca (L.L.); 3 Department of Chemistry, University of Alberta, Edmonton, AB T6G 2G2, Canada; 4 Department of Obstetrics and Gynaecology, Western University, B2-401 London Health Science Centre-Victoria Hospital, 800 Commissioners Rd E, London, ON N6H 5W9, Canada; 5 Children’s Health Research Institute, 800 Commissioners Rd E, London, ON N6C 2V5, Canada; 6 Lawson Health Research Institute, 750 Base Line Rd E, London, ON N6C 2R5, Canada

**Keywords:** BeWo cells, placental lipid metabolism, fatty acid desaturation, fatty acid elongation, transcriptome, metabolome, lipidome, OMIC integration

## Abstract

Maternal obesity and gestational diabetes mellitus (GDM) are linked with impaired placental function and early onset of non-communicable cardiometabolic diseases in offspring. Previous studies have highlighted that the dietary non-esterified fatty acids (NEFAs) palmitate (PA) and oleate (OA), key dietary metabolites associated with maternal obesity and GDM, are potential modulators of placental lipid processing. Using the BeWo cell line model, the current study integrated transcriptomic (mRNA microarray), metabolomic, and lipidomic readouts to characterize the underlying impacts of exogenous PA and OA on placental villous trophoblast cell metabolism. Targeted gas chromatography and thin-layer chromatography highlighted that saturated and monounsaturated NEFAs differentially impact BeWo cell lipid profiles. Furthermore, cellular lipid profiles differed when exposed to single and multiple NEFA species. Additional multi-omic analyses suggested that PA exposure is associated with enrichment in β-oxidation pathways, while OA exposure is associated with enrichment in anti-inflammatory and antioxidant pathways. Overall, this study further demonstrated that dietary PA and OA are important regulators of placental lipid metabolism. Encouraging appropriate dietary advice and implementing dietary interventions to maintain appropriate placental function by limiting excessive exposure to saturated NEFAs remain crucial in managing at-risk obese and GDM pregnancies.

## 1. Introduction

Maternal gestational obesity and gestational diabetes mellitus (GDM) are associated with adverse intrauterine environments that have been linked with an increased prevalence of pregnancy complications [1,2,3]. Offspring from these at-risk pregnancies often develop non-communicable cardiometabolic health disorders, including metabolic syndrome, obesity, and type 2 diabetes [4,5,6,7,8]. These children have been found to be impacted by these “adult-associated” metabolic diseases during early adolescence, highlighting the profound influence of the aberrant in utero programming resulting from maternal obesity and GDM [9,10,11]. Investigating the origins of these poor health outcomes in the exposed offspring is of great importance, as the rates of obesity and gestational diabetes mellitus (GDM) during pregnancy have been increasing globally over the past several decades [12,13]. It is currently estimated that one-third of pregnancies are impacted by maternal obesity and one-sixth of pregnancies are impacted by GDM, although these rates may be greater in some high-risk demographics [14,15,16]. The drastic increase in the prevalence of maternal obesity and GDM and subsequent increased rates of metabolic disease development in affected offspring will only create further strains on health care systems.

Aberrant placental metabolism in diabetic and obese pregnancies is an important factor underlying the in-utero programming of non-communicable cardiometabolic disorders [17,18,19]. These changes are thought to modulate placental nutrient metabolism and processing, ultimately leading to altered materno-fetal nutrient transport and adverse fetal growth conditions [19,20,21]. Specifically, impairments in placental mitochondrial respiration have been identified in term villous trophoblast cells from obese and GDM pregnancies that leads to reduced placental energy production and cellular ATP content [22,23,24,25,26]. Furthermore, maternal obesity has been linked with specific alterations in placental lipid processing at term, highlighted by increased expression and activity of key placental lipid transport proteins that, in conjunction with increased fatty acid desaturation and lipid esterification, is associated with placental triglyceride accumulation and steatosis [20,27,28,29]. Placental lysates from obese pregnancies have also shown a decreased abundance of short-chain acylcarnitine species (β-oxidation intermediaries) and reduced CPT1b expression, which indicates decreased placental β-oxidative activity. This suggests that the increased placental steatosis in obese pregnancies may be partly due to reduced placental lipid oxidation [20,30,31]. Furthermore, maternal GDM has been associated with increased placental steatosis at term that has likewise been attributed to increased lipid uptake and reduced placental β-oxidative activity [26,32,33,34,35].

Recently, studies have utilized “omic”-based research approaches to better identify and characterize the underlying biological mechanisms that facilitate placental dysfunction in these at-risk pregnancies [36,37]. Metabolomic and lipidomic analyses in particular have expanded our understanding of the mechanisms that facilitate impaired placental lipid processing. These omic-based readouts have demonstrated the presence of altered neutral and polar lipid profiles (highlighted by changes in underlying fatty acid (FA) composition) in the placentae from obese and GDM pregnancies [38,39,40,41,42]. In addition, pre-clinical mouse models of diet-induced maternal pre-gestational obesity have also described metabolomic and transcriptomic markers associated with altered placental lipid composition and metabolism [43,44]. Overall, the current literature has highlighted that abnormal lipid processing occurs in the obese and diabetic placenta. These alterations in placental metabolic function and lipid handling in obese and GDM pregnancies likely lead to suboptimal transplacental nutrient transport that could impact fetal development and increase the risk of metabolic disease in the exposed offspring [39,45].

Preceding the development of these dysfunctions in trophoblast metabolic function in obese and GDM pregnancies is an increased fat supply to the placenta due to maternal dyslipidemia [46,47]. Analysis of the fasting serum of women during the third trimester of pregnancy has revealed that conditions of GDM and obesity are associated with elevated circulating levels of non-esterified fatty acids (NEFAs) species [48]. These analyses also demonstrated that palmitate (C16:0 or FA 16:0; PA) and oleate (C18:1n9c or FA 18:1; OA) are the most abundant NEFA species in serum during pregnancy [48,49]. Since PA and OA are the most abundantly consumed FA in the diets of Westernized populations, these fats themselves may be an important link between poor maternal diet and impaired placental function in obese and GDM pregnancies [50,51,52,53]. Notably, maternal dietary FA consumption has previously been shown to be an important independent regulator of placental lipid handling [27,54]. Thus, it is likely the increased circulating levels of NEFAs in obesity and GDM directly facilitates the previously observed impairments in placental metabolism in these pregnancies. However, the extent to which the PA and OA directly modulate important placental lipid metabolic functions, such as lipid esterification, FA desaturation, and beta-oxidation, remains ill defined.

The purpose of the current study was to elucidate the underlying mechanisms that regulate placental lipid metabolism in response to elevated dietary fat supply. Hence, we examined the impacts of a prolonged (72 h) isolated dietary FA exposure using an in vitro cell culture-based system and BeWo trophoblast cells. First, we employed targeted lipidomic readouts to examine FA and neutral lipid profiles of BeWo progenitor cytotrophoblast (CT) and differentiated syncytiotrophoblast (SCT) cells. Our goals were to better understand how NEFA exposures affect placental lipid processing and to elucidate the impacts of NEFA exposures on placental FA desaturation and elongation processing. We postulated that exposure to elevated levels of dietary NEFA would alter BeWo trophoblast lipid profiles, which would be highlighted by increased triacylglycerol (TG) abundance and increased FA elongation and desaturation. Second, we used a transcriptomic mRNA microarray, in conjunction with untargeted metabolomic and lipidomic analyses, to explore the underlying mechanisms that regulate BeWo CT metabolism in response to an elevated dietary NEFA supply. Undifferentiated progenitor CT cells have previously been demonstrated to be the most metabolically active cells of the villous trophoblast layer, as well as the primary site of nutrient localization and storage in the placenta [26,55,56,57]. Thus, the current study sought to examine “omic” profiles in these more metabolically active CT cells. We postulated that dietary FA-treated BeWo CT cells would display altered transcriptomic, metabolomic, and lipidomic profiles indicative of altered lipid processing.

## 2. Materials and Methods

### 2.1. Materials

Materials for cell culture and targeted lipidomic (gas chromatography and thin-layer chromatography) procedures were purchased from Millipore Sigma (Oakville, ON, Canada) unless otherwise indicated. The solvents and reagents for untargeted lipidomics and metabolomics were acquired from Fisher Scientific (Hampton, NH, USA), Sigma-Aldrich (St. Louis, MO, USA), and Honeywell (Charlotte, NC, USA). Deuterated lipid standards for untargeted lipidomics were acquired from Avanti Polar Lipids (Alabaster, AL, USA).

### 2.2. Cell Culture Protocol

BeWo trophoblast cells (CCL-98) were purchased from the American Type Culture Collection (ATCC; Cedarlane Labs, Burlington, ON, Canada) and cultured in F12K media (Gibco, ThermoFisher Scientific, Mississauga, ON, Canada) as per ATCC guidelines. Cell culture media was supplemented with 10% Fetal Bovine Serum (Gibco) and 1% Penicillin-Streptomycin (Invitrogen, ThermoFisher Scientific, Mississauga, ON, Canada) for all experiments (complete F12K media). Cultures between passages 5–15 were utilized, and all cells were maintained at 5% CO_2_/95% atmospheric air for all collections. 

Cells were cultured with exogenous PA and OA, as previously reported [57]. In brief, BeWo trophoblast cells were plated onto cell culture dishes at the specific densities stated for each experiment and allowed to adhere to cell culture plates overnight prior to NEFA treatment. At T0H, cells were treated with complete F12K media further supplemented with either 100 µM PA, 100 µM OA, or 50 µM each PA and OA (P/O). All fatty acids were conjugated 2:1 (molar ratio) to Bovine Serum Albumin (BSA) to solubilize in cell culture media. Complete F12K media supplemented with BSA-alone (0.3%) was utilized as a control. Cell media was replenished every 24 h, and subsets of cells were additionally treated with 250 µM 8-Br-cAMP at T24H and T48H to induce CT-to-SCT differentiation. All cultures were collected for analysis after 72 h of NEFA exposure.

### 2.3. Fatty Acid and Neutral Lipid Profile Analysis of NEFA-Treated BeWo Trophoblasts

BeWo CT and SCT cells were plated at a density of 2 × 10^6^ cells in 150 mm cell culture dishes and cultured with exogenous NEFA species as described. At T72H, cells were collected via scrapping and washed twice with PBS, and the resulting cell pellet was flash frozen in liquid nitrogen and stored at −80 °C until analyzed.

Total cellular lipids were then collected from cell pellets via chloroform/methanol extraction (Folch extraction method) as previously reported and adapted for use with cell pellets [58,59,60]. In brief, cells were lysed in 2:1 chloroform:methanol with 0.01% butylated hydroxytoluene (BHT). Subsequently, 0.25% KCl was added, and samples were heated at 70 °C for 5 min to separate organic and aqueous layers. The organic layer was then collected and dried under a gentle stream of N_2_ gas. The collected lipids were resuspended in 2:1 chloroform:methanol (0.01% BHT) at 1 mg/mL concentrations and stored at −20 °C until analysis.

To examine fatty acid profiles, 100 µg of lipids were methylated to create fatty acid methyl esters (FAMEs), and subsequently separated and analyzed on a 6890 N gas chromatograph (Hewlett Packard, Palo Alto, CA, USA) with flame ionization detector (Agilent Technologies, Santa Clara, CA, USA) via a J&W Scientific High-Resolution Gas Chromatography Column (DB-23, Agilent Technologies; 30 m × 250 μm ID × 0.25 μm film thickness) as previously reported [59,60]. Separated FA species were then identified via comparison of retention times with known standards (Supelco 37 Component FAME Mix). The peak area of each fatty acid was quantified and expressed as a percent of total fatty acids detected.

Calculated fatty acid profiles were then utilized to quantify the activities of fatty acid desaturase enzymes by calculating desaturation indices, which reflect the ratio of enzyme product to substrate. Stearoyl-CoA Desaturase 1 (SCD1) activity was calculated as the ratio of 16:1n7/16:0, while 18:1n9/18:0; Fatty Acid Desaturase 1 (FADS1) activity was calculated as the ratio of 20:4n6/20:3n6; and Fatty Acid Desaturase 2 (FADS2) activity was calculated as the ratio of 18:3n6/18:2n6, as previously reported [61,62,63,64]. Additionally, FA elongation indices of 20:1n9/18:1n9; 18:0/16:0 and 22:5n3/20:5n3 were quantified to calculate the activity of FA elongase enzymes (reflective of enzyme product to substrate ratio) as previously reported in placental lysates [65].

Finally, the profiles of neutral lipid species in NEFA-treated BeWo trophoblasts were analyzed via Thin Liquid Chromatography coupled to Flame Ionization Detection (TLC-FID) as previously described [66]. In brief, collected lipid samples were dried under a gentle stream of N_2_ gas and resuspended in 100% chloroform. A total of 1–4 µL of lipid resuspensions were spotted onto TLC rods (Chromarod Type S5; Shell-USA, Spotsylvania, VA, USA) and developed in a TLC chamber with benzene:chloroform:formic acid (70:30:0.5 *v/v*/*v*). Chromatography rods were then dried and analyzed using the Iatroscan MK-6 TLC-FID system (Shell-USA). Peaks were identified via comparison with known standards, and neutral lipid peak area was quantified and expressed as a percentage of all neutral lipid species identified.

Percent fatty acid composition and percent neutral lipid composition, as well as FA desaturation and elongation index data, were analyzed via Randomized Block Two-Way ANOVA (2WA) and Tukey’s Multiple Comparisons Test. All statistical analysis was performed with GraphPad Prism v9.2.0 software (GraphPad Software, San Diego, CA, USA).

### 2.4. Transcriptomic Profiling of NEFA-Treated BeWo Cytotrophoblast Cells

BeWo CT cells were plated at a density of 3.5 × 10^5^ cells/well in 60 mm cell culture dishes and cultured with exogenous NEFAs, as described above. At T72H, the CT cells were washed once with PBS, collected in 0.9 mL TRIzol reagent (Invitrogen), and stored at −80 °C. Samples were then sent to the Genome Québec Innovation Centre for transcriptomic analysis using the Clariom S mRNA microarray (ThermoFisher Scientific, Mississauga, ON, Canada), as previously detailed [67]. In brief, RNA was automatically extracted using the QIAcube Connect (Qiagen, Toronto, ON, Canada). RNA concentration was determined using a Nanodrop 2000 (Nanodrop Technologies Inc., Wilmington, DE, USA), and RNA integrity was confirmed using a Bioanalyzer 2100 (Agilent Technologies, Waldbronn, Germany). cDNA was created from 100 ng total extracted RNA, fragmented, and labeled using the GeneChip WT Terminal Labeling Kit (ThermoFisher Scientific) following the manufacturer’s standard protocol. Labeled cDNA was hybridized to the Clariom S microarray chip using the GeneChip Hybridization oven 640 (Affymetrix, ThermoFisher Scientific, Mississauga, ON, Canada) for 17 h (60 rpm, 45 °C). The GeneChip Hybridization Wash and Stain Kit (ThermoFisher Scientific) was used to wash the microarray chips, and they were then scanned on a GeneChip scanner 3000 (ThermoFisher Scientific).

Microarray data were subsequently analyzed using Transcriptome Analysis Console v4.0 (ThermoFisher Scientific). Raw data were normalized using the Robust Multiple-Array Averaging (RMA) method. Each NEFA treatment condition was compared to the BSA control group in binary, and samples collected from the same passage were paired for analysis. Transcripts with ≥±1.3 fold-change (FC) vs. BSA control and raw *p*-value < 0.05 were determined to be differentially expressed.

To elucidate the impacts of gene expression changes in NEFA-treated BeWo CT cells, we next performed enrichment pathway analysis using both an over-representation analysis and Gene Set Enrichment Analysis (GSEA) approach. For over-representation enrichment analysis, each set of differentially expressed genes (DEGs) was imported into the WEB-based Gene SeT AnaLysis Toolkit (WebGestalt) for using the Wikipathways, Reactome, and Kyoto Encyclopedia of Genes and Genomes (KEGG) functional datasets, as well as the GeneOntology (GO) molecular function and biological processes datasets. Pathways with FDR-corrected *p*-value < 0.05 were considered significantly enriched. For the GSEA approach, all genes identified in the microarray panel were ranked according to signal-to-noise ratio using GSEA v4.2.3 software (Broad Institute Inc., Cambridge, MA, USA) [68,69]. The ranked gene lists were then uploaded to WebGestalt for GSEA analysis using the Wikipathways, Reactome, and KEGG functional datasets, as well as the GO molecular function and biological pathways datasets [70]. GSEA pathways with an FDR-correct *p*-value < 0.25 were determined to be significantly enriched in the NEFA-treated BeWo CT cells, as previously reported [69,71].

### 2.5. RT-qPCR Validation of Differentially Expressed Genes Identified by mRNA Microarray

Differentially expressed genes highlighted to be involved in lipid metabolic processes were selected for validation by RT-qPCR. RNA extracted by Genome Québec for mRNA microarray analysis was returned to our laboratory, and in conjunction with previously collected RNA (from an assessment of BeWo trophoblast syncytialization following NEFA exposure [57]), it was used for validation of DEGs. In brief, 2 µg of RNA was reverse transcribed using the High-Capacity cDNA Reverse Transcription Kit (Applied Biosystems; ThermoFisher Scientific, Mississauga, ON, Canada). RT-qPCR was subsequently performed using sample cDNA and the CFX384 Real Time System (Bio-Rad, Mississauga, ON, Canada). Relative expression of genes of interest was then determined using the ∆∆Ct method, with the geometric mean of PSMB6 and GAPDH utilized as a reference. The expression of genes of interest were analyzed in each NEFA treatment in binary versus BSA control samples via paired two-tailed *t*-test. The sequences of RT-qPCR primers utilized for microarray validation are available in Supplementary Table S1.

### 2.6. Cell Collections and Lysis for Untargeted Metabolomic and Lipidomic Profiling of BeWo Cytotrophoblast Cells

BeWo trophoblasts were plated at a density of 2 × 10^6^ cells/plate in 150 mm cell culture dishes and cultured with exogenous NEFAs, as described above. AT T72H, cell media were aspirated, and cells were washed three times with cold PBS. Cellular metabolism was then quenched with ice-cold methanol, and cells were scrapped and collected in microcentrifuge tubes. Samples were then dried first under a gentle stream of N_2_ gas, and residual moisture was removed via lyophilization. Samples were shipped to The Metabolomics Innovation Centre (TMIC, Edmonton, AB, Canada) for untargeted metabolomic and lipidomic profiling of NEFA-treated BeWo CT cells. The dried cell pellets were reconstituted in 400 µL of 1:1 (*v/v*) LC-MS grade methanol:water, and cells were lysed via five freeze–thaw cycles. Tubes were then centrifuged at 16,000× *g* for 10 min at 4 °C. A 300 µL aliquot of resulting supernatant was transferred to a fresh tube for metabolomic profiling, while the remaining supernatant and cell pellets were utilized for lipidomic profiling.

### 2.7. Untargeted Metabolomic Profiling of BeWo Cytotrophoblast Cells

Cellular metabolome profiles were analyzed using a High-Performance Chemical Isotope Labeling (HP CIL) liquid chromatography coupled mass spectrometry (LC-MS) approach, as previously described [67,72,73]. In brief, the 300 µL aliquot of supernatant was dried down and redissolved in water. A proprietary metabolome quantification kit from Nova Medical Testing Inc. (Product Number: NMT-6001-KT, Edmonton, AB, Canada) was used to measure the total metabolite concentration in each sample. Different volumes of each sample were taken and then dried down to adjust samples to the same total metabolite concentration of 2 mM. The samples were then chemically labeled for amine-/phenol-, hydroxyl-, carboxyl, and carbonyl- submetabolome profiling with dansylation reaction, base-activated dansylation reaction, DMPA bromide labeling, and dansylhydrazine labeling, respectively [74]. During labeling, individual samples were labeled with ^12^C-reagents, and a pooled sample, which was generated by mixing an aliquot from each individual sample, was labeled with ^13^C-reagents. The ^12^C-labeled samples were then mixed with the ^13^C-labeled pool in a 1:1 ratio. LC-MS was subsequently utilized for metabolome analysis using an Agilent 1290 LC with an Agilent Eclipse Plus reversed-phase C18 column (150 × 2.1 mm, 1.8 µm particle size) (Agilent Technologies, Santa Clara, CA, USA) linked to a Bruker Impact II QTOF Mass Spectrometer (Bruker Corporation, Billerica, MA, USA).

Metabolomics data were then imported into IsoMS Pro v1.2.7 (Nova Medical Testing Inc, Edmonton, AB, Canada) for data processing and analysis. Metabolites were identified in a three-tier approach with high-confidence identifications in tiers 1 (*m/z* and experimental retention time match to a library of labeled metabolite standards) and 2 (*m/z* and predicted retention time match), and putative matches in tier 3 (*m/z* match), as previously reported [74]. Metabolites with ≥±1.2 FC and raw *p*-value < 0.05 in each NEFA treatment versus BSA control were determined to be differentially abundant. Metabolite peak-pairs (i.e., ^12^C-labeled metabolite from each sample/^13^C-labeled metabolite from a pooled sample) identified in tiers 1 and 2 that had an associated KEGG pathway ID number were then imported into MetaboAnalyst v5.0 website for pathway enrichment analysis (using the homo sapiens KEGG Library) to determine biological impacts of differentially abundant metabolites. KEGG pathways with raw *p*-value < 0.05 were determined to be significantly enriched.

### 2.8. Integration of BeWo Cytotrophoblast Transcriptomic and Metabolomic Profiles

Peak pair data of metabolites in tiers 1 and 2 that have an associated KEGG ID number were subsequently imported into MetaboAnalyst v5.0, and metabolites with a ≥±1.2 FC and raw *p*-value < 0.05 in each NEFA treatment were identified. These differentially abundant metabolites were subsequently integrated with the list of DEGs identified in the Clariom S mRNA microarray using the Joint Pathway Analysis Tool in MetaboAnalyst v5.0 to determine enriched KEGG pathways. KEGG pathways with raw *p*-value < 0.05 that contained both DEGs and differentially abundant metabolites were determined to be significantly enriched integrated pathways.

### 2.9. Untargeted Lipidomic Profiling of BeWo Cytotrophoblast Cells

For lipidomic profiling, the remaining supernatant and cell pellets were processed using a modified Folch liquid–liquid lipid extraction protocol. In brief, samples were vortexed with 5 µL of Splash Lipidomix Mass Spec Standard (Avanti Polar Lipids, Alabaster, AL, USA) and methanol for 20 s, and subsequently vortexed for an additionally 20 s following the addition of dichloromethane. Samples were then incubated at room temperature for 10 min before being centrifuged at 16,000× *g* for 10 min at 4 °C. An aliquot of the resulting organic layer was evaporated to dryness with a SpeedVac. The resulting dried material was resuspended in 15 µL of mobile phase B (MPB, 10 mM ammonium formate in 95% isopropanol:water), vortexed for 1 min, and then diluted with 135 µL of mobile phase A (MPA, 10 mM ammonium formate in methanol:acetonitrile:water 50:40:10 *v*/*v*/*v*). A pooled mixture of all extracts was then prepared for quality control samples (QC) that were injected into the LC-MS system every 7 runs to monitor instrument performance. LC-MS analysis of the extracts was performed in both positive and negative ionization modes using a ThermoFisher Dionex UltiMate 3000 UHPLC (ThermoFisher Scientific) with a BEH C18 Column (Waters Corporation, Milford, MA, USA; 10 cm × 2.1 mm with 1.7 μm particles) coupled with a Bruker Impact II QTOF Mass Spectrometer (Bruker Corporation). Each sample was analyzed in technical duplicate. The chromatograms for the positive and negative ionization injections for each experimental replicate were aligned and combined into a single feature-intensity table using Metaboscope v4.0 (Bruker Corporation) [75,76].

A three-tier approach was utilized to identify the detected peaks. First, lipids were identified based on MS/MS spectral matches by searching the detected MS/MS spectra against the MS Dial LipidBlast database [77], the Human Metabolome database [78], and the MassBank of North America [79,80] LC-MS/MS libraries with Metaboscape v4.0. Compounds with MS/MS match score ≥ 500, precursor *m/z* error ≤ 5 mDa, and isotope pattern match (mSigma) score ≤ 50 were deemed tier 1 (high-confidence) identifications, while MS/MS match scores between 100 and 500 were considered tier 2 identifications. The employed automated identification routine embedded in Metaboscape 4.0 calculates a similarity score between the acquired spectrum and the spectra available in the employed databases in terms of *m/z* values of precursor and fragment ions and relative intensities. A higher MS/MS score corresponds to a higher proportion of matched fragments. Second, the unidentified lipid species were mass-matched to the LipidMaps database (*m/z* error ≤ 5 mDa) for tier 3 identifications. Since lipids can have many isomers and isobars within tight *m/z* tolerances, we employed a scoring approach to select the best identification candidate based on the expected performance of each lipid within our analytical platform (predicted retention time ranges, adduct form, predicted ionization efficiency and sensitivity, odd or even number of carbons in fatty acyl groups, and presence of unexpected modifications) [76].

For the untargeted lipidomics experiments, we followed the lipid nomenclature system suggested by the International Lipidomics Society. Tiers 1 and 2 identifications (MS/MS spectral match using similarity scores between the acquired spectra and common LC-MS/MS databases) were determined at the species or molecular species level, i.e., definition of lipid classes and subclasses, composition of fatty acyl/alkyl residues (or summed composition if individual residues are not specified in the source database), and functional groups. The position of double bonds and stereochemistry of compounds were not determined in this work. When provided, common names shown for selected lipids in tiers 1 and 2 were attributed based on biological intelligence rather than analytical evidence, i.e., the most common form of the molecule found in nature. Tier 3 identifications (*m/z* match using the LipidMaps databases) were determined at the species level, i.e., lipid class and subclass, total number of carbon atoms, total number of double bond equivalents, and total number of additional oxygen or other atoms. Although extensive, the identification routine employed in this work has limitations regarding isomeric or isobaric species. Lipids can have many isomeric forms with identical chemical formulas, masses, and MS/MS fragmentation patterns. The compounds may differ only in the position of double bonds, functional groups, or stereochemistry (e.g., sn positions for glycerol-based lipids). Although some of these lipids can produce distinct chromatographic peaks, they cannot be accurately identified at the structure-defined level by the employed untargeted LC-MS/MS approach. Hence, multiple peaks can be assigned as the same lipid at the molecular species or at the species level. Further information regarding the assumptions and definitions for lipid classification and nomenclature employed for this work can be found at Liebisch et al. (2020) [81].

The detected intensities for identified compounds were normalized to class-matches internal standards, i.e., a mixture of 14 deuterated lipids that represent the most abundant lipid classes in the known lipidome (Splash Lipidomix Mass Spec Standard, Avanti Polar Lipids). Unidentified features could not be matched to the most similar internal standard; hence, they were not employed for statistics. The normalized feature intensities (detected peak intensity/intensity of the most similar internal standard) were then imported to MetaboAnalyst v5.0 for statistical analysis. Non-informative features (i.e., near-constant features in samples based on RSD calculation) and features with low repeatability (RSD >30% in QC samples) were filtered out. The dataset was further normalized by the median intensity ratio within each experiment and auto-scaled. For univariate statistics using Volcano plots, lipid species with ≥±1.5 FC for NEFA treatment/BSA control and raw *p*-value < 0.05 were considered differentially abundant.

The normalized peak intensities of lipid species from each lipid class identified in tiers 1 and 2 were subsequently added together to determine the total abundance of each lipid class in the NEFA treatments. These summed data were imported into MetaboAnalyst v5.0 and the total lipid class intensities of each NEFA condition were compared in to the BSA control in binary using a Wilcoxon rank-sum test to determine differentially abundant lipid classes in each NEFA treatment. Significance was determined using a Bonferroni-corrected cut-off of *p* < 0.0167.

## 3. Results

### 3.1. NEFA-Treatments Impact Fatty Acid Profiles of BeWo Trophoblasts

A total of 10 saturated fatty acids (SFA) species, 6 mono-unsaturated fatty acid (MUFA) species, and 9 polyunsaturated fatty acid (PUFA) species were detected in the BeWo trophoblast samples. Palmitic acid (FA 16:0), stearic acid (18:0), oleic acid (18:1n9c), and arachidonic acid (20:4n6) were highlighted as the most abundant fatty acid species in BeWo trophoblast cultures (Table 1, *n* = 4–5/group).

Syncytialization of BeWo trophoblast cells was associated with significantly reduced relative abundances of 20:1n9 and 20:2, as well as significantly increased relative abundances of 18:2n6 and 20:4n6 compared to undifferentiated BeWo cells (Table 1; *p* < 0.05, *n* = 4–5/group).

PA-treated BeWo cultures displayed significantly increased relative abundances of total SFA species, 16:0, 16:1n7, 22:2, and 20:5n3, as well as significantly decreased relative abundances of total MUFA species, 18:0, and 18:1n9c in both CT and SCT cultures relative to the respective differentiation state BSA-control (Table 1; *p* < 0.05, *n* = 4–5/group). PA-treated BeWo CT cells further displayed a significantly increased relative abundance of 22:5n3, while PA-treated BeWo SCT cells displayed significantly reduced relative abundances of 18:1n7 and 18:2n6 versus respective BSA-control cultures (Table **1**; *p* < 0.05, *n* = 4–5/group).

The OA-treatment in BeWo CT and SCT trophoblasts was associated with significantly increased relative abundance of total MUFA species, 18:1n9c, and 20:1n9, as well as a significantly reduced relative abundance of total SFA and PUFA species, 18:0, 24:0, 16:1n7, 18:1n7, 16:3n4, 18:2n6, 20:3n6, 20:4n6, and 22:5n3 versus respective differentiation state BSA-control cultures (Table 1; *p* < 0.05, *n* = 4–5/group). Additionally, OA-treated BeWo CT cells displayed significantly decreased relative abundances of 14:0, and 16:0, while OA-treated BeWo SCT cells displayed a significantly reduced relative abundance of 18:3n6 versus the respective differentiation state BSA-control (Table 1; *p* < 0.05, *n* = 4–5/group).

*p*/O-treated BeWo CT and SCT cells were found to have increased relative abundances of total MUFA species, 20:1n9, and 20:2, as well as decreased relative abundances of 18:0, 24:0, 18:1n7, 18:2n6, and 20:3n6 compared to BSA-control cultures (Table 1; *p* < 0.05, *n* = 4–5/group). P/O-cultured BeWo CT cells further demonstrated an increased relative abundance of 18:1n9c, as well as decreased relative abundance of total SFA species 14:0, 16:1n7, and 16:3n4 versus BSA-control CT cultures (Table 1; *p* < 0.05, n = 4–5/group). Additionally, P/O-treated BeWO SCT cells displayed a decreased relative abundance of 18:3n6 compared to BSA-control SCT cultures (Table 1; *p* < 0.05, n = 4–5/group).

### 3.2. The Impact of NEFA Treatments on Desaturation and Elongation Indices in BeWo Trophoblasts

**Figure 1:**
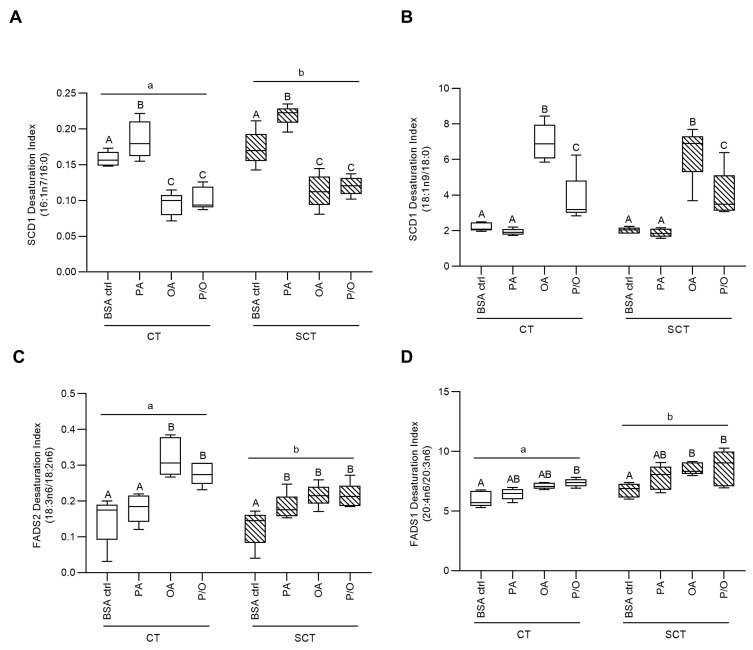
Fatty Acid Desaturase Enzyme Activity Indices in NEFA-treated BeWo CT and SCT cells. The abundance of desaturase enzyme substrate and product FAs were quantified via Gas Chromatography coupled to Flame Ionization Detection, and the activity of each desaturase enzyme was quantified via desaturation index, reflecting the ratio of enzyme product to substrate. The Stearoyl-CoA Desaturase-1 (SCD1) index was quantified via the ratios (**A**) 16:1n7/16:0 ratio and (**B**) 18:1n9/18:0; (**C**) the Fatty Acid Desaturase 1 index was quantified via the ratio of 18:3n6/18:2n6; and (**D**) the Fatty Acid Desaturase 2 index was quantified via the ratio of 20:4n6/20:3n6 (*n* = 5/group; different upper-case letters denote statistical significance between NEFA treatments within each BeWo differentiation state (CT or SCT cells), and different lower-case represents statistical significance between differentiation state groups (CT and SCT cells); *p* < 0.05, Two-Way Randomized Block ANOVA, Tukey’s multiple comparisons Test).

Fatty acid profile data were utilized to quantify the activity of fatty acid desaturase and elongase enzymes via calculation of the ratios of enzyme products to substrates (desaturation/elongation index). OA and P/O-treated BeWo SCT and SCT cells displayed significantly reduced SCD1 activity compared to respective BSA-control cultures when assessed by the 16:1n7/16:0 index (Figure 1A; *p* < 0.05, *n* = 5/group). However, when SCD1 activity was assessed by the ratio of 18:1n9c/18:0, OA- and P/O-treated BeWo CT and SCT cells were found to have increased SCD1 activity compared to respective BSA-control cultures (Figure 1B; *p* < 0.05, *n* = 5/group). Furthermore, OA-treated BeWo CT cells displayed increased FADS2 activity (Figure 1C; *p* < 0.05, *n* = 5/group) while OA-treated SCT cells displayed increased FADS2 and FADS1 activity compared to BSA-control cultures (Figure 1C,D; *p* < 0.05, *n* = 5/group). P/O-treated BeWo CT and SCT also displayed increased FADS1 and FADS2 activity compared to BSA-controls (Figure 1C,D; *p* < 0.05, *n* = 5/group). Finally, PA-treated BeWo SCT cells displayed increased FADS2 activity compared to BSA-control SCT cells (Figure 1C; *p* < 0.05, *n* = 5/group). BeWo syncytialization was additionally linked with increased SCD1 activity (when assessed as 16:1n7/16:0) and FADS1 activity, as well as decreased FADS2 activity (Figure 1A,C,D; *p* < 0.05, *n* = 5/group).

**Figure 2:**
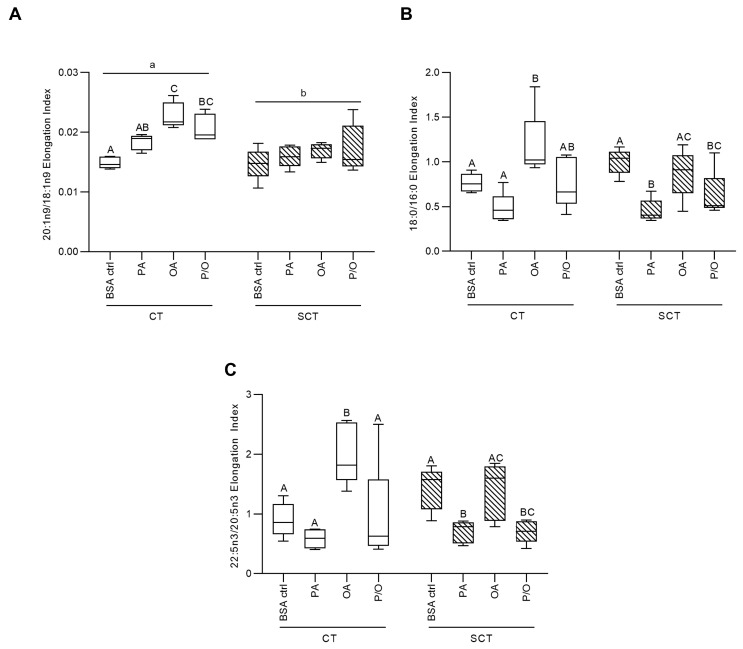
Fatty Acid Elongase Enzyme Activity Indices in NEFA-treated BeWo CT and SCT cells. The abundance of products and substrates of FA elongase enzymes were quantified via Gas Chromatography Coupled to Flame Ionization Detection. The ratios of (**A**) 20:1n9/18:1n9, (**B**) 18:0/16:0, and (**C**) 22:5n3/20:5n3 (elongation indices) were quantified to determine elongase enzyme activities (*n* = 5/group; different upper-case letters denote statistical significance between NEFA treatments within each BeWo differentiation state (CT or SCT cells), and different lower-case represents statistical significance between differentiation state groups (CT and SCT cells); *p* < 0.05, Two-Way Randomized Block ANOVA, Tukey’s multiple comparisons).

OA-treatment was additionally associated with increased elongation of 18:1n9, 16:0, and 20:5n3 in BeWo CT cells, while P/O-treated BeWo CT were only observed to have increased 18:1n9 elongation (Figure 2A–C). PA-treatment in BeWo SCT cells, however, was linked with reduced 16:0 and 20:5n3 elongation (Figure 2B,C). BeWo SCT also displayed an overall reduced elongation of 18:1n9 compared to undifferentiated BeWo CT cells (Figure 2A).

### 3.3. OA-Treatment Alters Neutral Lipid Profiles of BeWo Trophoblasts

Subsequent TLC-FID analysis of extracted lipids from BeWo cultures identified five neutral lipid species in all samples. Specifically, the analyses identified cholesterol ester (CE), free fatty acids (FA), triglycerides (TG), free cholesterol, and diacylglycerols (DG) in BeWo trophoblast neutral lipid fraction. OA-treated BeWo CT and SCT displayed increased relative triglyceride abundance, as well as decreased relative free cholesterol abundance relative to respective differentiation state BSA-control cultures (Table 2; 2WA: NEFA treatment *p* < 0.05, *n* = 5/group).

### 3.4. Transcriptomic Profiles of BeWo Cytotrophoblasts Are Impacted by NEFA Treatment

**Figure 3:**
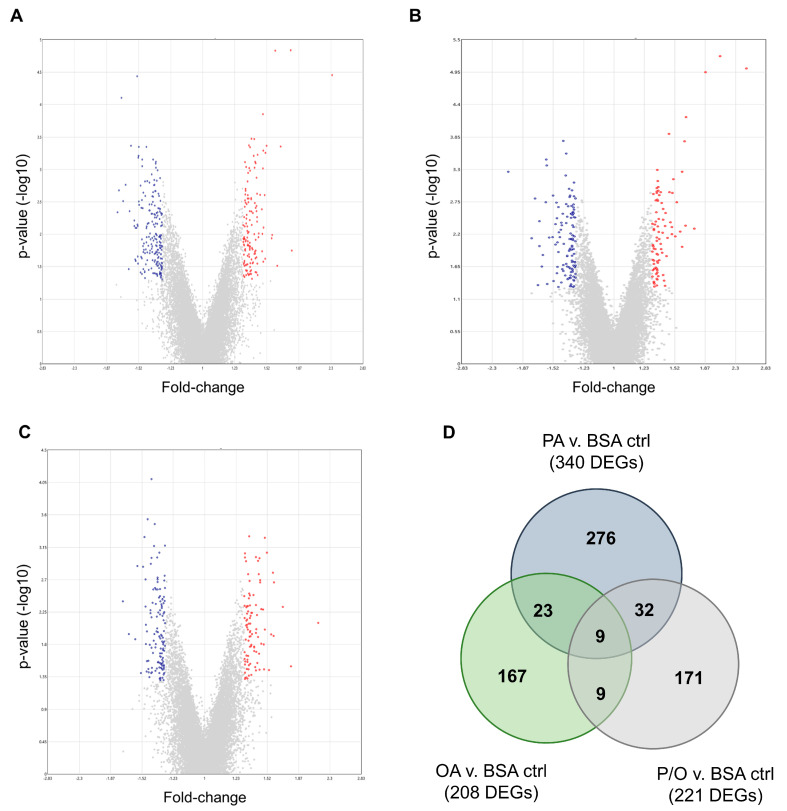
Volcano plot visualization of differentially expressed genes in BeWo CT cells cultured with 100 µM NEFAs for 72 h. The transcriptome profiles of NEFA-treated BeWo cytotrophoblast cells was determined via Clariom S mRNA microarray, and differentially expressed genes were determined with ± 1.3 fold-change and raw-*p* < 0.05 cut-offs. Volcano plots were constructed to visualize the differentially expressed genes (DEGs) in NEFA-treated BeWo CT cells. (**A**) PA-treated cells displayed 340 DEGs (140 upregulated and 200 downregulated); (**B**) OA-treated cells displayed 208 DEGs (88 upregulated and 120 downregulated); and (**C**) P/O-treated cells displayed 221 DEGs (96 upregulated and 125 downregulated) compared to BSA-alone treated cells (*n* = 5/group). The x-axis of the volcano plots indicates fold-change vs. BSA control, and the y-axis indicates *p*-value (−log_10_). Red dots represent upregulated genes, and blue dots represent downregulated genes. (**D**) A Venn diagram showing the number of differentially expressed genes that are unique to each NEFA-treatment or common to multiple NEFA-treatments.

PA-treated BeWo CT cultures displayed 340 differentially expressed genes (DEGs) (140 upregulated and 200 downregulated); OA-treated CT cultures displayed 208 DEGs (88 upregulated and 120 downregulated); and P/O-treated BeWo CT cultures displayed 221 DEGs (96 upregulated and 125 downregulated) when compared to BSA-control CT cultures (≥± 1.3 FC vs. BSA control CT cultures; raw-*p* < 0.05, *n* = 5/group). A summary of the DEGs identified in each NEFA-treatment condition is available in Supplementary Table S2. Volcano plots were then constructed to visualize differentially expressed transcripts in each NEFA-treatment (Figure 3A–C).

A Venn diagram was additionally constructed to visualize the DEGs that were unique to each NEFA-treatment, as well as those common in two or all three of the NEFA-treatment conditions (Figure 3D). Twenty-three differentially expressed transcripts were found to be common between PA and OA-treated CT cells; thirty-two DEGs were common between PA and P/O treatments; and nine genes were found to be differentially expressed in both OA and PO-treated cells (Figure 3D). A further 9 genes were found to be differentially expressed in all NEFA-treated BeWo CT cells (Figure 3D). A summary list of unique and common DEGs is available in Supplementary Table S3.

Over-representation analysis revealed no significantly enriched pathways in the DEGs lists in PA- and OA-treated BeWo CT cells (Supplementary Table S4). In the P/O-treated BeWo CT cells, there was a significant enrichment in the Wikipathways “Fatty Acid Biosynthesis” dataset (associated with the DEGs: *ACACB* ((+1.32 FC), *ACSL5* (+1.77 FC), *ACSL6* (−1.34 FC) and *SCD* (−1.24 FC)), as well as in the GO “CXCR3 Chemokine Receptor Binding” molecular function dataset (associated with the DEGs: *CXCL10* (+1.39 FC), *CXCL13* (+1.42 FC), and *CXCL11* (−1.37 FC)) (Supplementary Table S4, FDR-*p* < 0.05).

Subsequent enrichment analysis using a GSEA approach highlighted a significant downregulation of the Reactome “Adherens Junctions Interactions” pathway (associated with the DEGs: *CDH17* (−1.48 FC), and *CDH9* (−1.36FC)) in PA-treated BeWo CT cells (Supplementary Table S5, FDR-*p* < 0.25). In PA-treated BeWo CT cells, a significant upregulation was also observed in the Reactome “Peptide Hormone Biosynthesis” pathway (no associated DEGs), as well as the KEGG datasets for: “Staphylococcus Aureus Infection” (associated with the DEG: *ITGB2* (+1.4 FC)); “PPAR Signaling Pathway” (associated with the DEGs: *ACSL5* (+1.77 FC), *APOA2* (+1.51 FC), *FABP2* (+1.31 FC), *FABP6* (+1.36 FC), *HMGCS2* (+1.34 FC), and *PLIN2* (+1.35 FC)); “Th1 and Th2 Cell Differentiation” (associated with the DEGs: *IKBKG* (+1.32 FC), and *IL12A* (+1.35 FC)); “Fatty Acid Biosynthesis” (associated with the DEGs: *ACSL5* (+1.77 FC), and *ACACB* (+1.57 FC)); “Leishmaniasis” (associated with the DEGs: *ITGB2* (+1.4 FC), *NCF1* (+1.35 FC), and *IL12A* (+1.35 FC)); “Cortisol Synthesis and Secretion” (associated with the DEGs: *CREB3L3* (+2.31 FC), *CYP21A2* (+1.31 FC), and *HSD3B1* (+1.34 FC)); “Ovarian Steroidogenesis” (associated with the DEGs: *CYP1A1* (+1.37 FC), and *HSD3B1* (+1.34 FC)); “Adipocytokine Signaling Pathway” (associated with the DEGs: *ASCL5* (+1.77 FC), *ACACB* (+1.57 FC), and *IKBKG* (+1.32 FC)); and “Primary Bile Acid Biosynthesis” (no associated DEGs) (Supplementary Table S5, FDR-*p* < 0.25)

In the OA-treated BeWo CT cells, there was a significant upregulation in the Wikipathways “PPAR Signaling Pathway” dataset (associated with the DEG: *ACSL5* (+1.87 FC)) and in the KEGG “PPAR Signaling Pathway” dataset (Associated with the DEGs: *ACSL5* (+1.87 FC), and *PLIN2* (+1.46 FC)) (Supplementary Table S5, FDR-*p* < 0.25). A significant downregulation was also observed in KEGG “Linoleic Acid Metabolism” dataset (no associated DEGs) in OA-treated BeWo CT cells (Supplementary Table S5, FDR-*p* < 0.25).

In the P/O-treated cells, a significant upregulation was observed in the Reactome “Gene and protein expression by JAK-STAT signaling after Interleukin-12 stimulation” pathways (no associated DEGs), as well as in the GO “Amine Transport” biological processes dataset (no associated DEGs) (Supplementary Table S5, FDR-*p* < 0.25). Summary Ordered lists of the genes identified in the microarray panels, and their respective signal-to-noise ratio scores, are available in Supplementary Table S6.

### 3.5. RT-qPCR Validation of Differentially Expressed Genes

**Figure 4:**
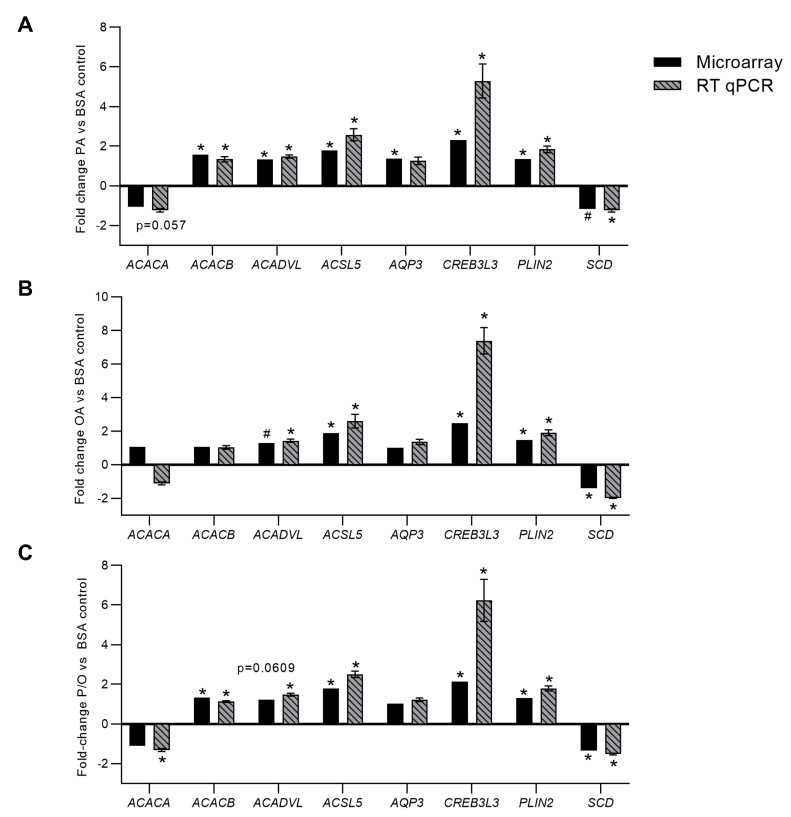
RT-qPCR validation of differentially expressed genes in NEFA-treated BeWo CT cells. Differential expression of genes involved in lipid metabolic processes were validated in (**A**) PA-, (**B**) OA-, and (**C**) P/O-treated BeWo CT cells via RT-qPCR. RT-qPCR data were analyzed via paired two-tailed *t*-test (*n* = 9/group), and the data expressed as fold-change vs. BSA-control cultures (mean ± SEM). * indicates ≥ ±1.3 FC vs. BSA-control, *p* < 0.05 for microarray data; and *p* < 0.05 for RT-qPCR data. ^#^ indicates <1.3 FC vs. BSA-control, *p* < 0.05 for microarray data.

In PA-treated BeWo CT cells, RT-qPCR analysis confirmed differential expression of *ACACB* (+1.57 FC microarray; +1.34 FC RT-qPCR); *ACADVL* (+1.32 FC microarray; +1.47 FC RT-qPCR); *ACSL5* (+1.77 FC microarray; +2.57 FC RT-qPCR); *CREB3L3* (+2.31 FC microarray; +5.29 FC RT-qPCR); *PLIN2* (+1.35 FC microarray; +1.83 FC RT-qPCR) (Figure 4A, *p* < 0.05, *n* = 9). It is important to note that *SCD* mRNA abundance although statistically significant (*p* < 0.05) in the microarray panel did not meet the ±1.3 FC cut-off to be considered differentially expressed in PA-treated CT cells (−1.16 FC). Subsequent RT-qPCR analysis likewise highlighted a significant decrease in *SCD* mRNA abundance in PA-treated BeWo CT cells (−1.24 FC; Figure 4a, *p* < 0.05, *n* = 9). The microarray panel also highlighted a significant downregulation in a transcript best identified as *ACACA*, however RT-qPCR analysis demonstrated no significant alterations in *ACACA* mRNA abundance (Figure 4A, *p* = 0.057, *n* = 9). Finally, *AQP3* was highlighted as differentially expressed in the microarray panel, however there was no significant difference in *AQP3* mRNA abundance when assessed by RT-qPCR (Figure 4A, *p* = 0.4287, *n* = 9).

In OA-treated BeWo CT cells, RT-qPCR analysis confirmed the differential expression of: *ACSL5* (+1.87 FC microarray; +2.60 FC RT-qPCR); *CREB3L3* (+2.48 FC microarray; +7.38 FC RT-qPCR); *PLIN2* (+1.46 FC microarray; +1.90 FC RT-qPCR); and *SCD* (−1.41 FC microarray; −1.98 FC RT-qPCR) (Figure 4B, *p* < 0.05, *n* = 9). The microarray panel also highlighted a significant increase in *ACADVL* mRNA abundance although this transcript did not meet the ±1.3 FC cut-off to be considered differentially expressed. However, it is important to note that RT-qPCR analysis also highlighted a significant increase in *ACADVL* mRNA abundance in OA-treated BeWo CT cells (+1.43 FC; Figure 4B, *p* < 0.05, *n* = 9).

In P/O-treated BeWo CT cells, RT-qPCR analysis confirmed the differential expression of *ACACB* (+1.32 FC microarray; +1.12 FC RT-qPCR); *ACSL5* (+1.77 FC microarray; +2.50 FC RT-qPCR); *CREB3L3* (+2.13 FC microarray; +6.23 FC RT-qPCR); *PLIN2* (+1.30 FC microarray; +1.79 FC RT-qPCR); and *SCD* (−1.34 FC microarray; −1.52 FC RT-qPCR) (Figure 4C; *p* < 0.05, *n* = 9/group). The RT-qPCR analysis additionally highlighted a significant increase in the expression of *ACADVL* (+1.48 FC) and a decrease in the expression of *ACACA* (−1.32 FC) (Figure 4C, *p* < 0.05, *n* = 9/group). Interestingly, the expression of both *ACADVL* (−1.22 FC, *p* = 0.0609) and *ACACA* (−1.11 FC, *p* = 0.0862) were trending towards significance in the microarray panel.

### 3.6. Metabolomic Profiles of BeWo Cytotrophoblast in Response to NEFA Treatment

**Figure 5:**
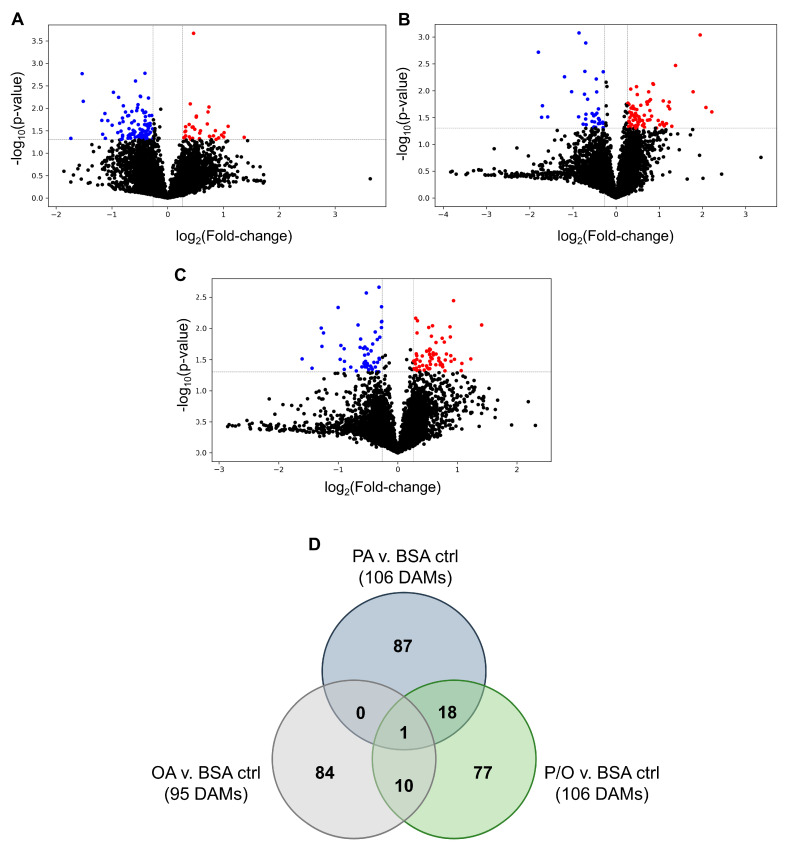
Differentially abundant metabolites in NEFA-treated BeWo CT cultures. Volcano plots were constructed to visualize differentially abundant metabolites (DAMs) in NEFA treated BeWo CT cells. (**A**) PA-treated BeWo CT cells displayed 106 DAMs (32 increased and 74 decreased); (**B**) OA-treated cells displayed 95 DAMs (55 increased and 40 decreased); and (**C**) P/O-treated BeWo CT cultures displayed 106 DAMs (56 increased and 50 decreased) compared to BSA-control cultures (≥±1.2 fold-change, raw-*p* < 0.05). The x-axis of the volcano plots represents log_2_ (fold-change) vs. BSA-control, and the y-axis represents *p*-value (−log_10_). Red dots represent individual metabolites with significantly increased abundance, and blue dots represent metabolites with significantly reduced abundance. (**D**) A Venn diagram showing the number of differentially abundant metabolites that were unique to each NEFA-treatment or common to multiple NEFA-treatments.

On average, 6569 ± 50 (mean ± SD) metabolite features were identified in each NEFA-treated BeWo CT sample. A summary of the identified features and metabolite peak-pair data are available in Supplementary Table S7. Of these features, 179 metabolites were identified with high confidence in tier 1 (*m/z* and retention time match to an experimental library of metabolite standards), and a further 602 metabolites were identified with high confidence in tier 2 (*m/z* and predicted retention time match). In PA-treated cultures, 2 metabolites in tier 1 (both decreased); 7 metabolites in tier 2 (4 increased and 3 decreased); and 97 metabolites in tier 3 (*m/z* match, 28 increased and 69 decreased) were found to be differentially abundant when compared to BSA-control samples (≥±1.2 FC, raw-*p* < 0.05, *n* = 5/group). Furthermore, 5 metabolites identified in tier 1 (2 increased and 3 decreased); 12 metabolites from tier 2 (2 increased, and 10 decreased); and 78 metabolites in tier 3 (51 increased, and 27 decreased) were found to be differentially abundant in OA-treated CT cells when compared to BSA-control samples (≥±1.2 FC, raw-*p* < 0.05, *n* = 5/group). Finally, 7 metabolites identified in tier 1 (4 increased and 3 decreased); 7 metabolites identified in tier 2 (4 increased and 3 decreased); and 92 metabolites identified in tier 3 (48 increased and 44 decreased) were found to be differentially abundant in P/O-treated BeWo CT cultures when compared to BSA-control samples (≥±1.2 FC, raw-*p* < 0.05, *n* = 5/group). Volcano plots were constructed to visualize the differentially abundant metabolite species in each NEFA-treatment group versus BSA-control samples (Figure 5A-C). An unsupervised principal component analysis (PCA) 2D plot as well as supervised partial-least squares discriminant analysis (PLS-DA) 2D plot were constructed to visualize the degree of difference between metabolite profiles in all NEFA-treatment groups (Supplementary Figure S1). Summary lists of the differentially abundant metabolites identified in each NEFA condition are available in Supplementary Table S8.

A Venn diagram was constructed to visualize the number of unique and shared differentially abundant metabolites in the NEFA treatments which highlighted that most differentially abundant metabolites were unique to one NEFA treatment (Figure 5D). Only 3 high confidence identified metabolites (from tiers 1 and 2) were found to be differentially abundant in at least 2 of the NEFA treatments. Specifically, the abundance of Kahweol was found to be significantly increased (+1.69 FC in OA; +1.64 FC in P/O); the abundance of N-acetylethanolamine was found to be significantly decreased in both the OA and P/O treatments (−1.66 FC in OA; −1.46 FC in P/O); and the abundance of 3-hydroxymethylglutaric acid was found to be significantly decreased in both the PA and P/O treatments (−2.89 FC in PA; −2.00 FC in P/O). Summary lists of the differentially abundant metabolites common between 2 or more NEFA-treatment groups is available in Supplementary Table S9.

**Figure 6:**
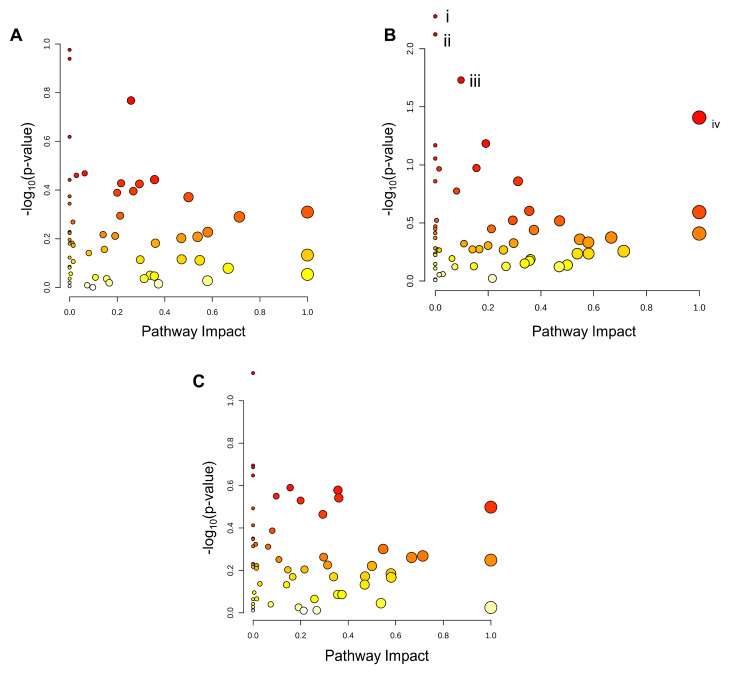
Pathway Analysis of metabolite profiles of NEFA-treated BeWo CT cells. Metabolite peak pairs identified in tier 1 and tier 2 were imported into MetaboAnalyst v5.0 for analysis of enriched KEGG pathways. Pathways with raw-*p* < 0.05 were considered significantly enriched, and scatterplots were created for (**A**) PA-treated; (**B**) OA-treated; and (**C**) P/O-treated cultures to visualize identified KEGG pathways. The x-axis of the scatterplot represents pathway impact, and y-axis represents pathway enrichment *p*-value (−log_10_). Each individual node represents a unique KEGG pathway, and node size corresponds to pathway impact, while node color corresponds to the *p*-value of the pathway. The KEGG pathways for (i) Ether lipid metabolism; (ii) Amino sugar and nucleotide sugar metabolism; (iii) Fructose and mannose metabolism; and (iv) Taurine and hypotaurine metabolism were highlighted to be significantly enriched in the OA-treated BeWo CT cultures.

Analysis of KEGG pathway enrichment of the metabolite sets from tiers 1 and 2 was subsequently performed using MetaboAnalyst software. Scatterplots were then constructed to visualize the KEGG pathway nodes highlighted by each metabolite set (Figure 6A–C). No KEGG pathways were identified as significantly enriched in the metabolite sets from PA- and P/O-treated BeWo CT cells (Figure 6A,C). The KEGG pathways for (i) Ether lipid metabolism (highlighted by increased sn-glycerol-3-phosphoethanolamine (+3.44 FC) levels), (ii) Amino sugar and nucleotide sugar metabolism (highlighted by reduced 6-deoxy-L-galactose (−1.17 FC) levels); (iii) Fructose and mannose metabolism (highlighted by reduced 6-deoxy-L-galactose (−1.17 FC) levels); and (iv) Taurine and hypotaurine metabolism (highlighted by a trend towards reduced abundance of 5-L-Glutamyl taurine (−1.82 FC; *p* = 0.062) and 3-sulfino-L-alanine (−1.47 FC; *p* = 0.054)) were determined to be significantly enriched in the OA-treated CT metabolite set (raw-*p* < 0.05; Figure 6B).

### 3.7. BeWo Cytotrophoblast Transcriptome and Metabolome Integration

**Figure 7:**
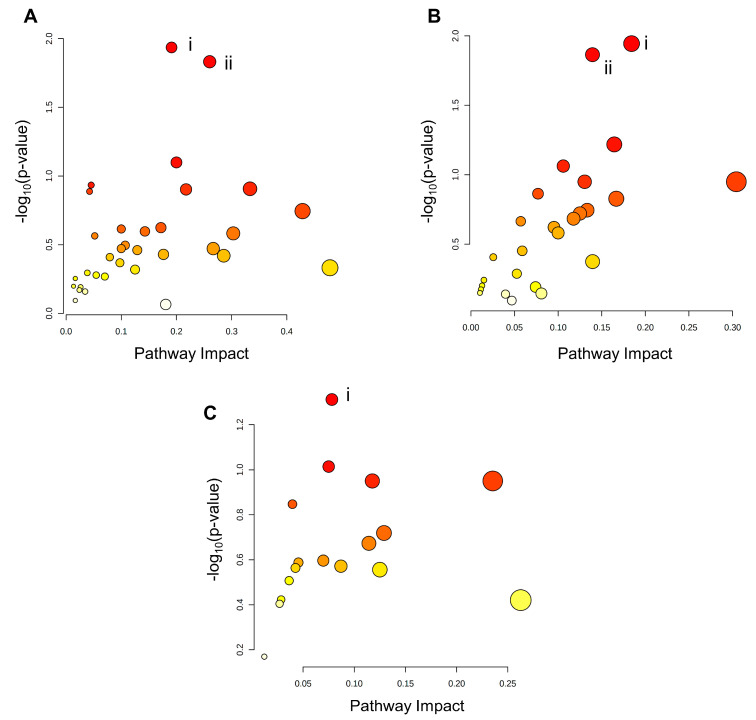
Joint Pathway Analysis of Transcriptome and Metabolome Profiles of NEFA-treated BeWo CT cells. Differentially expressed gene and differentially abundant metabolite (from identification tiers 1 and 2) datasets were analyzed via the Joint Pathway Analysis module in MetaboAnalyst Software. Pathways with raw-*p* < 0.05 were considered significantly enriched, and scatterplots were constructed to visualize identified KEGG pathways. The x-axis of the scatterplot represents pathway impact, and y-axis represents pathway enrichment *p*-value (−log_10_). Each individual node represents a unique KEGG pathway, and node size corresponds to pathway impact, while node color corresponds to the *p*-value of the pathway. (**A**) The (i) inositol phosphate metabolism and (ii) phosphatidylinositol signaling system KEGG pathways were highlighted as significantly enriched in PA-treated BeWo CT cells. (**B**) The (i) ether lipid metabolism and (ii) purine metabolism KEGG pathways were highlighted as significantly enriched in the OA-treated BeWo CT cells. (**C**) The (i) fatty acid biosynthesis KEGG pathway was highlighted as significantly enriched in P/O-treated BeWo CT cells.

Integration of the transcriptomic and metabolomic datasets revealed no significantly enriched pathways in the KEGG database containing both differential genes and differential metabolites in the PA- and P/O-treated BeWo CT cells (Figure 7A,C). However, the integration analysis highlighted significantly enriched pathways only containing DEGs in the PA and P/O treatments. Specifically, there was a significant enrichment in the (i) Inositol Phosphate Metabolism and (ii) Phosphatidylinositol Signaling System KEGG pathways (both highlighted by downregulation of IMPA1 (−1.34 FC) and PIP5K1B (−1.37 FC) as well as upregulation of PLCE1 (+1.50 FC) and INPP5D (+1.447 FC)) in the PA-treated BeWo CT cells. We also noticed a significant enrichment in the (i) Fatty Acid Biosynthesis KEGG Pathway (highlighted by upregulation of ACACB (+1.32 FC) and ACSL5 (+1.77 FC) and downregulation of ACSL6 −1.34 FC)) in the P/O-treated BeWo CT cells (raw-*p* < 0.05; Figure 7A,C).

Integration analysis of the OA-treated BeWo CT cell transcriptome and metabolome profiles highlighted significantly enriched pathways containing both differential genes and metabolites. Specifically, the (i) Ether Lipid Metabolism pathway (highlighted by increased accumulation of sn-glycero-3-phosphoethanolamine (+3.44 FC) in conjunction with upregulation of GDPD1 (+1.34 FC) and downregulation of PLA2G12A (−1.35 FC) and (ii) Purine Metabolism pathway (highlighted by increased levels of adenosine (+3.30 FC), inosine (+2.45 FC), and guanosine (+1.69 FC), in conjunction with upregulation of PRPS1 (+1.31 FC), and ADK (+1.31 FC), as well as downregulation of GMPR (−1.76 FC) were found to be significantly enriched in OA-treated BeWo CT cells (raw-*p* < 0.05; Figure 7B).

### 3.8. The Impact of NEFA-Treatment of BeWo Cytotrophoblast Lipidome Profiles

An average of 9199 ± 295 (mean ± SD) lipid species were identified in each sample. Of these features, 950 were identified in tier 1 (MS/MS similarity score ≥500 with *m/z* and isotopic pattern match), 161 were identified in tier 2 (MS/MS similarity score between 100 and 500 with *m/z* and isotopic pattern match), and a further 6808 features were identified in tier 3 (*m/z* match only). A summary of the identified features is available in Supplementary Table S10, and a list of lipid class abbreviations is available in Supplementary Table S11.

**Figure 8:**
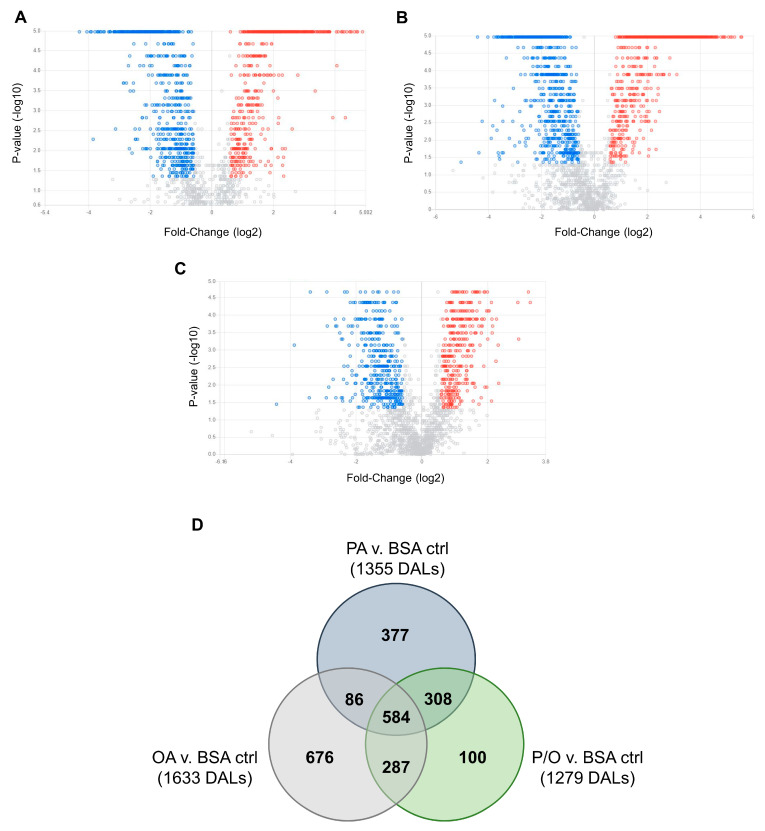
Volcano Plot visualization of differentially abundant lipid species in NEFA-treated BeWo CT cells. Differentially abundant lipid species (DALs) were visualized via non-parametric volcano plot. The x-axis of the volcano plot represents log_2_ (fold-change), and the y-axis represents *p*-value (−log_10_). Lipid species with significantly increased abundance vs. BSA-control cultures are represented by red dots, and lipid species with significantly decreased abundance are represented by blue dots (≥±1.5 fold-change, raw *p*-value < 0.05). (**A**) PA-treated BeWo CT cells displayed 1355 differentially abundant lipids (676 increased and 679 decreased); (**B**) OA-treated BeWo CT cells displayed 1633 differentially abundant lipids (799 increased and 834 decreased); and (**C**) P/O-treated BeWo CT cells displayed 1279 differentially abundant lipids (639 increased and 640 decreased). (**D**) A Venn diagram showing the number of DALs unique to each NEFA-treatment and shared among two or more NEFA-treatments.

Non-parametric volcano plots were then constructed to visualize differentially abundant lipid species in each NEFA-treatment compared to BSA-control samples (Figure 8; FC (NEFA-treatment / BSA control ≥±1.5; raw-*p* < 0.05). In PA-treated samples, 190 lipid species in tier 1 (80 decreased and 110 increased); 28 lipid species in tier 2 (12 increased, and 16 decreased); and 1137 lipid species in tier 3 (553 increased, and 584 decreased) were found to be significantly altered (Figure 8A). In OA-treated samples, 197 lipid species in tier 1 (73 increased, and 124 decreased); 30 lipid species in tier 2 (15 increased; and 15 decreased); and 1406 lipid species in tier 3 (711 increased and 695 decreased) were found to be significantly altered (Figure 8B). In P/O-treated BeWo CT cells, 163 lipid species in tier 1 (55 increased, and 108 decreased); 24 lipid species in tier 2 (9 increased, and 15 decreased); and 1092 lipid species in tier 3 (575 increased; and 517 decreased) were found to be significantly altered (Figure 8C). A summary of the altered lipid species in each NEFA condition is available in Supplementary Table S12.

A Venn diagram was constructed to visualize the number of differentially abundant lipid species (DALs) that were unique to a single NEFA-treatment and shared between multiple NEFA treatments (Figure 8D). In total, 377 DALs were unique to PA-treated BeWo CT cells, 676 DALs were unique to OA-treated BeWo CT cells, and 100 DALs were unique to P/O-treated BeWo CT cells. Additionally, 86 lipid species were altered in both PA and OA-treated cells, 308 lipid species were altered in both PA- and P/O-treated cells, and 287 lipid species were altered in both OA and P/O-treated BeWo CT cells. Finally, it was found that 584 lipid species were differentially abundant in all NEFA treatment groups. A summary of the lipid species unique to a single NEFA condition or shared between two or more groups is available in Supplementary Table S13.

An unsupervised principal component analysis (PCA) 2D plot as well as supervised partial least squares discriminant analysis (PLS-DA) 2D plot were constructed to visualize the degree of difference between lipidome profiles in all NEFA-treatment groups and highlighted a separation in lipid profiles between the different NEF treatments (Supplementary Figure S2).

**Figure 9:**
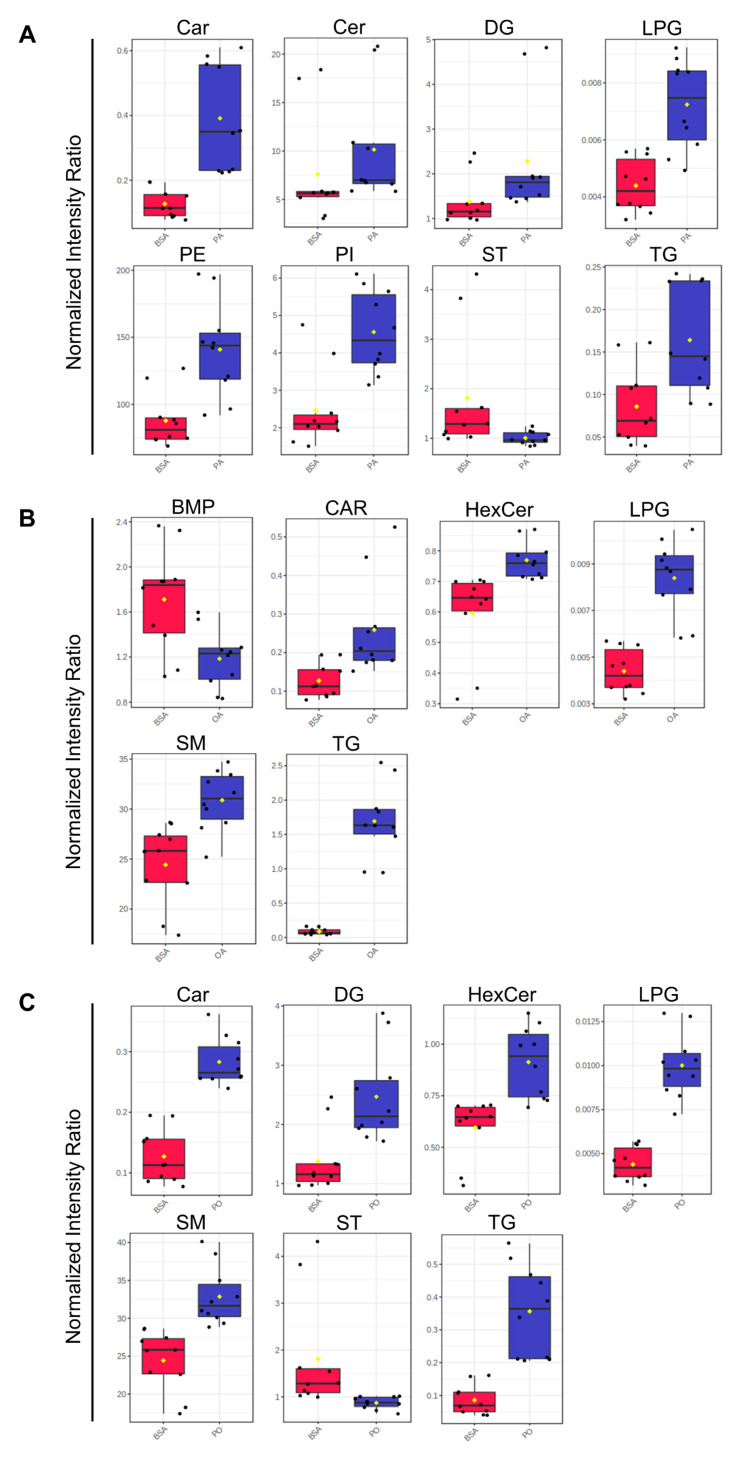
Differentially Abundant Lipid Classes in NEFA-treated BeWo CT cells. Differentially abundant lipid species identified in tiers 1 and 2 were sorted by lipid sub-class, and total peak intensity of lipids in each class was determined. The abundances of lipid class in each NEFA treatment were compared to the BSA-control samples in binary via Wilcoxon rank sum test. Boxplots were constructed to visualize lipid classes with differential abundance versus BSA-control in (**A**) PA-treated, (**B**) OA-treated, and (**C**) P/O-treated BeWo CT cells (raw-*p* < 0.0167). The red boxplots in each figure represent the lipid-class abundance in the BSA-control BeWo CT cells, and the blue boxplots in each figure represent the lipid-class abundance in the respective NEFA-treated BeWo CT cells.

Analysis of the totaled peak intensities of lipids from each independent class revealed eight differentially abundant lipid classes in PA-treated BeWo CT cells; six differentially abundant lipid classes in OA-treated BeWo CT cell; and seven differentially abundant lipid classes in P/O-treated BeWo CT cells (Figure 9A–C; raw-*p* < 0.0167). Specifically, the abundance of lipids in the Ceramide (CER), Phosphatidylethanolamine (PE), and Phosphatidylinositol (PI) classes were only significantly increased in PA-treated BeWo CT cells. The lipids in the Bis[monoacylglycero]phosphate (BMP) class were only significantly decreased in OA-treated BeWo CT cells. No significantly altered lipid classes were unique to the P/O treatment group. The Fatty Acyl Carnitine (Car), Lysophosphatidylglycerol (LPG) and Triacylglycerol (TG) lipid classes were found to be significantly elevated in all NEFA treatment groups relative to BSA control. The Diacylglycerol (DG) class was significantly increased, and the cholesterol and derivatives (ST) lipid class was significantly decreased in both the PA and P/O treatments. Finally, the Glucosylceramide (HexCer) and Ceramide-1-phosphate (SM) lipid classes were found to be significantly elevated in both the OA and P/O treated BeWo CT cells.

## 4. Discussion

PA and OA are the most abundant circulating NEFA species in the serum of pregnant women, and the concentrations of these NEFA are reportedly elevated under conditions of maternal GDM and obesity [48]. Dietary fat consumption data have highlighted that PA and OA are the most widely consumed dietary fats in Westernized cultures [50,51,52,53]. Since the maternal diet has been demonstrated to be an important regulator of placental metabolic function [27,54], these fats themselves may be important in regulating placental function in obese and GDM pregnancies. The current study utilized a multi-omics research approach to characterize the independent and combined impacts of the dietary NEFA species PA and OA on placental lipid metabolic processing using BeWo villous trophoblast cells.

Previously, BeWo trophoblast cells have been found to be functionally, metabolically, and morphologically similar to primary human trophoblast cultures [57,82,83]. Overall, these similarities highlight the utility of the BeWo cell culture system in modeling human trophoblast responses to isolated elevations in dietary nutrients. Notably, the use of a cell line system as reported here is not confounded by variables that influence primary cell culture systems (such as gestational age, maternal gestational diet, and delivery method [84,85]) and allowed the independent analysis of NEFA-mediated placental responses. Further, the 100 µM concentrations of NEFAs utilized in this study were reflective of physiological fasting levels of PA and OA during the third trimester of gestation [48], and more importantly, our research group has previously demonstrated that they do not impact BeWo trophoblast viability or differentiation capacity at 72 h [57]. Thus, the data presented in the current study also highlights alterations in placental lipid processing independent from lipotoxic effects. In addition, this previous work by our research group has identified that these NEFA doses impact functional readouts of BeWo CT mitochondrial function, further highlighting their relevance.

Our first objective was to utilize targeted lipidomic analyses via GC-FID and TLC-FID to characterize cellular FA and neutral profiles, as well as FA desaturation and elongation indices to better understand lipid processing in BeWo CT and SCT cells cultured with dietary NEFA species. We subsequently utilized a multi-omics research approach (combining transcriptomic with untargeted metabolomic and lipidomic analyses) to characterize the underlying biochemical impacts of an increased NEFA supply on the metabolic function of BeWo CT cells, given that previous studies have highlighted the dominant metabolic profile of these progenitor placental trophoblast cells [26,55,56,57]. Overall, our results highlighted that the dietary NEFAs PA and OA have different metabolic fates in placental trophoblast cells, as well as that these FAs are processed differentially when exposed to trophoblasts in isolation (PA and OA treatments) and in combination (P/O treatments).

### 4.1. Impacts of Dietary NEFA on BeWo FA and Neutral Lipid Profiles

The current study utilized GC-FID as a preliminary readout of the impacts of an increased dietary NEFA supply on BeWo trophoblasts via analysis of cellular FA profiles [38,39,40]. Specifically, the current study highlighted that a 72-h PA exposure was associated with increased SFA levels in both CT and SCT cultures, with specific elevations in the C16:0 content in both differentiation states. OA exposure, however, resulted in a profound increase in cellular MUFA species, and strikingly, C18:1n9 accounted for almost two-thirds of all FA content in both OA-treated CT and SCT cells. In contrast, the P/O treatments only moderately impacted BeWo SFA and PUFA compositions, and no specific alterations in C16:0 composition were observed in either the P/O-treated CT or SCT cells. However, the P/O-treated cells displayed altered MUFA profiles and increased C18:1n9 levels similar to the trends in the OA treatments, although with a lower magnitude of change. Interestingly, previous data from rodent models have highlighted that placental MUFA (and not SFA) content correlates with maternal plasma FA levels [86], similar to the findings in P/O-treated BeWo cells reported here. Overall, these data highlighted that altering the supply of dietary NEFA to placental trophoblasts independently impacts the trophoblast cell FA profile and suggests that increased circulating NEFA levels contribute to alterations to placental lipid compositions. Additionally, these data highlight that dietary NEFA differentially impact trophoblast FA profiles when elevated independently and in combination with other dietary fats, suggesting that both the quantity and composition of fats is important in regulating placental lipid processing. It is important to note that altered FA compositions have previously been described in placentae from obese and GDM pregnancies [38,39,40], as well as in the development of placental dysfunctions, including preeclampsia [87]. Thus, specific dietary NEFA-induced alterations to FA profiles reported here may reflect a transition towards aberrant placental function in the BeWo cultures.

The collected FA profile data also allowed for investigation into elongation and desaturation metabolic processing of dietary FA through the calculation of FA elongase and desaturase indices [61,62,63,64,65]. Of particular interest in the current study was FA desaturation mediated by Stearoyl-CoA Desaturase 1 (SCD1), which is responsible for the production of palmitoleate (POA; C16:1n7) and OA (C18:1n9) from PA (C16:0) and stearate (C18:0), respectively. Previously, obesity has been found to impact placental FA desaturation via SCD1, although current reports have been somewhat inconsistent. Specifically, placentae of obese pregnancies have been found to display an increased placental abundance of SCD1 mRNA, suggesting increased FA desaturation [20]. A subsequent report, however, described an overall decrease in SCD1 activity in placentae of obese pregnancies without alterations in SCD protein abundance, highlighting that changes in FA desaturation may also be regulated by post-translational modifications [88]. In the current study, we observed decreased SCD1 activity (as assessed by the POA/PA ratio) in OA- and P/O-treated BeWo CT and SCT cells. These data could indicate that previously observed reductions in SCD1 activity in term placentae of obese pregnancies could be facilitated in part by an increased placental OA or MUFA supply [88]. In contrast, PA-treated BeWo CT and SCT cells displayed an elevated SCD1 index (as assessed by the POA/PA ratio). As POA has been linked with increased oxygen consumption and β-oxidation activity in adipocytes [89,90], we speculate that increased PA desaturation to POA in PA-treated BeWo CT cells may underlie the increased mitochondrial respiratory activity that our research group has previously observed in these cells [57].

Another measure of placental lipid processing of interest to the current study is the desaturation mediated through the enzyme Fatty Acid Desaturase 2 (FADS2). FADS2 is responsible for the synthesis of long-chain polyunsaturated FA species [91], and its expression is altered in the placentae of some pre-eclamptic pregnancies [92]. Our results demonstrated that OA-exposure, both alone and in combination with PA, resulted in increased FADS2 production of γ-Linoleic Acid (GLA; C18:2n6), as well as increased elongation of OA to Gondoic Acid (C20:1n9). Increased levels of these specific FA species have previously been linked to anti-inflammatory outcomes [93,94]. As OA has been demonstrated to attenuate the lipotoxic effects of increased PA levels in trophoblast cells [95,96], we speculate that OA partially exerts its protective effects on trophoblast cells via the increased production of anti-inflammatory lipid intermediaries.

Overall, our data demonstrated that modulating the dietary FA composition impacts placental FA content, as well as FA elongation and desaturation processing. However, the overall impact that these changes in placental FA composition and desaturation ultimately have on placental lipid handling and transplacental nutrient transport remains poorly understood. Future studies utilizing radiolabeled NEFA species and transwell culture systems, as previously described [83,97], are still needed to fully characterize the impacts of altered placental lipid content and identify the specific mechanisms that link dysfunctional placental lipid metabolism to the development of cardiometabolic diseases in the exposed offspring.

Using TLC-FID, the current study also demonstrated that the proportion of TG in the neutral lipid fractions of BeWo CT and SCT cells was elevated only in cells exposed to OA independently, likely reflecting that OA is highly lipogenic in trophoblasts. These results were consistent with previous reports in cultured placental explants and isolated trophoblasts [95,98] and NEFA-treated BeWo [83,99] cells that have demonstrated that imported OA is highly localized to TG lipid fractions. This may indicate that an increased supply of OA to the placenta is an important factor that helps facilitate the increased TG synthesis that has previously been reported in obese and GDM placentae [20,26,28,32]. Interestingly, the P/O-treated BeWo CT and SCT cells were not observed to have altered neutral lipid and TG compositions when assessed by TLC-FID. These data aligned with reports from isolated primary human trophoblasts, demonstrating reduced lipid droplet formation with a combined OA and PA exposure compared to OA-alone exposures [95]. Subsequent analysis of lipid class abundance using untargeted LC-MS/MS also highlighted increased levels of PI and PE lipid species in PA-treated BeWo CT cells. These results were also consistent with readouts in primary placental trophoblasts (PHTs) that have highlighted PA is highly localized to trophoblast phospholipid fractions [88]. These data may indicate further similarities in lipid processing in PHT and BeWo trophoblast cell culture systems. Overall, this suggests that saturated and monounsaturated dietary NEFA have different metabolic fates in placental trophoblasts, as well as that the metabolic fates of these FA species are altered when present in combination with other dietary FAs.

The FA and neutral lipid profile data, however, highlighted limited alterations in trophoblast lipid profiles following syncytialization, and, specifically, only four differentially abundant FA species were found in BeWo SCT cells. In contrast, previous work in cultured primary human trophoblasts (PHTs) cells demonstrated profound reductions in the levels of many FA species in placental villous trophoblast cells following syncytialization [100]. The differences between the current study and previous reports are likely due to differences in the reporting of FA profile data and may highlight a limitation in the current study. The current study reported FA profiles as a percentage of total FA abundance, which contrasts with previous reports that highlighted the protein-normalized FA quantity. Thus, the current study was not able to report whether BeWo trophoblast cells demonstrate similar reductions in the absolute quantities of different FA species, as was highlighted with PHT cultures [100]. Future works using quantitative readouts of FA abundances are therefore needed to establish if BeWo trophoblasts exhibit similar differences in lipid metabolism between differentiated and progenitor CT cells, as well as differentiated SCT cells.

### 4.2. A Multi-Omics Analysis of NEFA-Treated BeWo CT Metabolic Function

The multi-omic analyses revealed that exposure to different dietary NEFA species extensively altered BeWo CT cellular transcriptome and lipidome profiles but led to limited alterations in cellular metabolome profiles. The mRNA microarray transcriptomic analyses highlighted 340 DEG in PA-treated BeWo CT cells and 308 DEGs in OA-treated BeWo CT cells, as well as 221 DEGs in P/O-treated BeWo CT cells. Subsequent over-representation enrichment analysis highlighted one significantly enriched functional pathway in the transcriptomic datasets. Specifically, the Wikipathways “Fatty Acid Biosynthesis Pathway” was significantly enriched in P/O-treated BeWo CT cells. Further enrichment analysis using a GSEA approach further highlighted alterations in lipid signaling and metabolic pathways in the PA OA-treated BeWo CT cells. For example, PA-treated cells displayed a significant upregulation of genes involved in the Reactome “Fatty Acid Biosynthesis” and “PPAR Signaling Pathway” datasets, while OA-treated cells displayed a significant upregulation in the Wikipathways and KEGG “PPAR Signaling Pathway” datasets, as well as a significant downregulation in the KEGG “Linoleic Acid Metabolism” dataset. Overall, pathway enrichment analysis of NEFA-treated BeWo cells transcriptomic profiles demonstrated an altered regulation of key genes involved in lipid metabolic pathways. This data further indicates that placental trophoblasts modulate lipid processing functions in direct response to an increased supply of PA and OA.

Differentially expressed genes involved in lipid metabolism processes were identified, and their expression patterns were validated by RT-qPCR. The microarray readouts highlighted an upregulation of Acyl-CoA Synthetase Long Chain Family Member 5 (*ACSL5*) mRNA, as well as perilipin 2 (*PLIN2*) mRNA, in all NEFA-treated BeWo CT cells. Acyl-CoA synthetase (ACSL) enzymes have previously been highlighted to be directly involved in FA uptake and are responsible for conjugating FA species to Coenzyme A, an important first step in lipid metabolism that helps prevent FA efflux [88,101,102]. Interestingly, ACSL5 expression has also been found to be induced in PUFA-exposed BeWo trophoblasts, leading to an overall increase in FA uptake [103]. The data from the current study therefore suggests that ACSL5 is also involved in the processing of PA and OA in BeWo trophoblasts, and its increased expression likely facilitates increased cellular uptake of FA species. PLIN2, on the other hand, has been shown to be required for lipid droplet synthesis in trophoblasts [104]. More importantly, placental expression of PLIN2 has been demonstrated to be elevated in GDM pregnancies [105], and its expression has been found to be correlated with maternal pre-pregnancy Body-Mass-Index (BMI) [28]. Thus, the current study suggests that an increased supply of dietary NEFAs to the placenta in obese and GDM pregnancies may directly facilitate this increased expression of PLIN2. In turn, this dietary NEFA-mediated modulation of PLIN2 expression may promote TG accumulation in lipid droplets and subsequently placental steatosis in obese and GDM pregnancies.

Both ACSL5 and PLIN2 were DEGs involved in the enriched “PPAR signaling pathway” in both PA- and OA-treated BeWo CT cells. These genes are known to be specifically induced by PPARγ [106,107], suggesting that PPARγ is activated in placental trophoblasts in response to elevated NEFA levels. However, all NEFA-treated BeWo CT cells also displayed a reduced mRNA abundance of SCD1, another lipid metabolism gene that has been shown in some tissue types to be induced by PPARγ [108,109]. The reduced expression of SCD1 in the current study could indicate that certain lipid metabolism genes are differentially regulated by PPARγ in placental trophoblast compared to what has previously been shown in other tissues. Notably, PPARγ itself is regulated by multiple post-translational modifications (PTMs), including phosphorylation, SUMOylation, acetylation, and ubiquitination [110]. NEFA-mediated alterations in these PTMs could explain why some PPAR-associated genes were activated and others were repressed in our BeWo trophoblast cells. Characterizing these modifications in future investigations will be important to fully understand the impacts of NEFAs on placental metabolism.

The microarray analysis further highlighted an increase in the mRNA abundance of cAMP responsive element-binding protein 3-like 3 (CREB3L3) in all NEFA treatment groups. CREB3L3 is a transcription factor that has been demonstrated to regulate lipid metabolism via modulating the expression of genes involved in FA oxidation [111,112,113]. Interestingly, CREB3L3 has also been implicated in the pathophysiology of inflammation and Endoplasmic Reticulum (ER) stress [112,113,114]. This could indicate that CREB3L3 controls lipid processing functions in trophoblasts, as well as underlies the development of inflammation and ER stress that has previously been observed in some PA-treated placental trophoblasts [95,96,115]. The microarray analysis in the current study however, revealed that BeWo CT cells exposed to 100 µM levels of PA for 72 h do not have elevated expression of genes related to inflammation (such as TNFα, IL6, and IL-32), and ER stress (such as BCL2, DDIT3, and XBP1). In contrast, these inflammation and ER stress pathways have previously been shown to be induced in PA-treated PHT cultures [95,115]. These differential responses may arise from variations in the PA dose utilized (100 µM in the current study compared to 200–500 µM in primary trophoblasts [95,115]). We speculate that the increased CREB3L3 expression highlighted in the current study may reflect an early timepoint in transition towards ER stress and inflammation in PA-exposed BeWo trophoblasts.

Subsequently, the current study utilized an integrative approach combining readouts from transcriptomic, metabolomic, and lipidomic datasets to further describe the impacts of dietary NEFA exposure on BeWo CT metabolic function. Specifically, the transcriptome and metabolome datasets were integrated via the Joint Pathway Analysis module of the MetaboAnalyst software. This analysis highlighted a significant enrichment in the Purine Metabolism pathway in OA-treated BeWo CT cells that was associated with increased cellular accumulations of adenosine (+3.30 FC), inosine (+2.45 FC), and guanosine (+1.69 FC) that may be suggestive of reduced purine breakdown. Increased degradation of purines has previously been shown to be involved in placental responses to increased inflammation [116]. The increased purine accumulation in OA-treated trophoblasts shown here may further suggest that anti-inflammatory processes are activated in dietary OA-treated trophoblasts. Alternatively, the increased purine abundances, in conjunction with the upregulation of phosphoribosyl pyrophosphate synthetase 1 (PRPS1) observed in the microarray panel, could reflect increased purine synthesis via the Pentose Phosphate Pathway (PPP), which has previously been shown to operate in placental tissues [117,118,119]. As the PPP increases the production of NADPH, a metabolite with antioxidant effects [120,121], these data may highlight that OA also acts to reduce oxidative stress in placental trophoblasts.

Combining readouts from the transcriptomic, metabolomic, and lipidomic datasets also highlighted a potential shift in oxidative substrate selection in BeWo trophoblasts cultured with PA (both alone and in conjunction with OA) towards increased FA oxidation (β-oxidation). Examination of differentially abundant metabolites in PA- and P/O-treated BeWo CT revealed a significant reduction in intracellular levels of 3-hydroxymethylglutaric acid (−2.89 FC in PA-treated CT cells; −2.00 FC in P/O-treated CT cells), a byproduct of leucine degradation [122]. We speculate that this may indicate a reduced breakdown of branched-chain amino acid species into byproducts that can enter the Tricarboxylic Acid Cycle (TCA Cycle) in BeWo trophoblasts cultured under increased PA levels. Additionally, we observed increased C16:0 carnitine (+13.92 FC in PA-treated CT cells; +3.79 FC in P/O-treated CT cells) levels in BeWo trophoblasts treated with PA. As FA species must first be conjugated to carnitine before being translocated to the mitochondrial matrix for oxidation [123], the increased acylcarnitine levels in the cells may reflect a metabolic shift towards using PA as an oxidative fuel.

Moreover, PA- and P/O-treated BeWo CT cells were found to have elevated expression of genes involved in β-oxidative metabolism. Specifically, we observed increased expression of Very-Long Chain Acyl-CoA Dehydrogenase (*ACADVL*), the enzyme responsible for the catalyzing the preliminary step in β-oxidation metabolism, and an increased expression of Acetyl-CoA Carboxylase Beta (*ACACB*), an enzyme thought to control β-oxidative activity by catalyzing the carboxylation of acetyl-CoA to malonyl-CoA. Notably, elevated β-oxidative activity has previously been linked with increased generation of Reactive Oxygen Species (ROS) [124,125], which ultimately promotes oxidative mitochondrial damage [126,127]. Therefore, the PA-mediated increases in β-oxidation associated genes and metabolites in BeWo trophoblasts could be indicative of an early transition towards placental mitochondrial dysfunction.

Interestingly, malonic acid, an end-point metabolic byproduct of β-oxidation that is synthesized by ACACB, was only found to be increased in P/O-cultured BeWo CT cells (+1.85 FC), and not in PA-cultured cells. As malonate has been found to limit β-oxidation activity by inhibiting acylcarnitine transport into the mitochondrial matrix [128], these data may highlight that BeWo trophoblasts exposed to PA in combination with OA, but not PA-alone, are able to restrict excessive β-oxidation activity. Malonate-mediated inhibition of β-oxidation in the P/O-cultured BeWo CT cells may act to suppress excessive mitochondrial ROS production and could therefore limit mitochondrial membrane damage. We additionally speculate that these data may highlight a shift towards incomplete β-oxidation in BeWo trophoblasts cultured with PA alone, as these cells (but not P/O-treated CT cells) also displayed increased intracellular levels of the shortened-chain C14:0 acylcarnitine (+3.99 FC). Increased production of short-chain acylcarnitine species has previously been liked with pro-inflammatory processes [129] and mitochondrial overload [130], and thus, these data may further highlight a transition towards inflammation and mitochondrial dysfunction in PA-exposed trophoblasts. However, the insights of the current study into incomplete β-oxidation are minimized due to the limited identifications of short-chain acylcarnitine species in our untargeted lipidomic readouts. Future studies may need to utilize targeted metabolomic approaches or utilize radiolabeled FA species to better identify the production of short-chain acylcarnitine species and incomplete β-oxidation in PA-treated BeWo trophoblasts.

### 4.3. Conclusions

The results of the current study highlighted the continued utility of the BeWo cell line as a pre-clinical model of the human placenta, as well as that the dietary FAs PA and OA are important regulators of placental trophoblast lipid metabolism. Overall, BeWo cells treated with PA displayed elevated saturated FA content, as well as increases in markers associated with elevated β-oxidative activity and incomplete β-oxidation. In contrast, BeWo cells treated with OA displayed elevated triglyceride content and increases in markers associated with anti-inflammatory and antioxidant pathways. These results support the notion that increased levels of certain dietary fats in maternal circulation are involved in facilitating the aberrant placental function in obese and GDM pregnancies, but more importantly, demonstrate that saturated and unsaturated FA species differentially impact placental function.

It is important to note, however, that the use of a cell line model system in the current study may limit the clinical translation and implication of our findings. While a cell line model is useful in its ability to examine the isolated impacts of dietary nutrients, these independent increases are not fully reflective of the complex environment in which the human placental is maintained in vivo. Future studies are still needed to elucidate the combined impacts of excess PA and OA with additional dietary nutrients, including PUFAs and glucose. Likewise, further examination of the impacts of dietary NEFAs on placental metabolism using primary cell culture systems and representative animal models are needed to fully elucidate placental responses to dietary-mediated adverse environments.

Overall, the data presented here suggests that an increased supply of dietary NEFAs leads to aberrant placental nutrient processing. These changes may ultimately impact transplacental nutrient transport and facilitate pathological conditions that predispose the exposed offspring to develop cardiometabolic health complications. Monitoring circulating FA levels in obese and GDM pregnancies, and subsequently implementing dietary interventions through modulating dietary fat content and composition, may be necessary in the clinical management of these at-risk pregnancies. Implementation of healthy diet and lifestyle choices that reduce placental NEFA exposures in these “at-risk” pregnancies could act to maintain placental metabolic function throughout gestation and, in turn, reduce the risk of future metabolic health complications in the offspring.

## Figures and Tables

**Table 1:** Fatty acid profiles (% mol) of NEFA-treated BeWo trophoblast cells.

Fatty Acid Species	CT				SCT				Differentiation State Difference
	BSA Ctrl	PA	OA	P/O	BSA Ctrl	PA	OA	P/O	
ΣSFA	39.64 ± 1.1 *a*	44.91 ± 1.63 *b*	20.73 ± 1.04 *c*	33.28 ± 2.99 *d*	37.20 ± 0.92 *a*	44.67 ± 0.85 *b*	24.32 ± 3.18 *c*	34.25 ± 3.29 *a*	NS
8:0	0.63 ± 0.23	0.32 ± 0.11	0.40 ± 0.16	0.54 ± 0.10	0.49 ± 0.14	0.34 ± 0.12	0.45 ± 0.11	0.45 ± 0.06	NS
10:0	0.40 ± 0.14	0.31 ± 0.09	0.37 ± 0.11	0.37 ± 0.11	0.21 ± 0.07	0.24 ± 0.05	0.30 ± 0.07	0.21 ± 0.05	NS
14:0	1.43 ± 0.35 *a*	0.85 ± 0.31 *ab*	0.52 ± 0.12 *b*	0.74 ± 0.25 *b*	1.03 ± 0.33	0.68 ± 0.24	0.40 ± 0.08	0.58 ± 0.18	NS
15:0	0.54 ± 0.16	0.51 ± 0.19	0.19 ± 0.03	0.34 ± 0.14	0.45 ± 0.10	0.59 ± 0.19	0.28 ± 0.02	0.48 ± 0.14	NS
16:0	19.73 ± 0.81 *a*	27.84 ± 2.12 *b*	8.27 ± 0.81 *c*	17.52 ± 2.87 *a*	16.32 ± 0.78 *ab*	28.07 ± 1.37 *c*	12.17 ± 3.01 *a*	19.74 ± 2.80 *b*	NS
18:0	14.98 ± 0.48 *a*	12.79 ± 0.76 *b*	9.23 ± 0.41 *c*	12.04 ± 0.79 *b*	16.20 ± 0.36 *a*	12.46 ± 0.89 *b*	9.26 ± 0.41 *c*	10.98 ± 0.43 *bc*	NS
20:0	0.78 ± 0.06	0.94 ± 0.09	0.79 ± 0.16	0.74 ± 0.07	1.21 ± 0.17	1.10 ± 0.24	0.73 ± 0.10	0.92 ± 0.14	NS
22:0	0.68 ± 0.11	0.80 ± 0.16	0.47 ± 0.10	0.53 ± 0.06	0.76 ± 0.11 *ab*	0.91 ± 0.08 *a*	0.47 ± 0.07 *b*	0.74 ± 0.08 *ab*	NS
23:0	0.49 ± 0.14	0.58 ± 0.11	0.52 ± 0.02	0.64 ± 0.05	0.23 ± 0.03	0.44 ± 0.12	0.27 ± 0.03	0.41 ± 0.09	NS
24:0	1.02 ± 0.05 *a*	1.08 ± 0.07 *a*	0.70 ± 0.06 *b*	0.79 ± 0.07 *b*	0.98 ± 0.05 *a*	0.89 ± 0.05 *a*	0.54 ± 0.02 *b*	0.63 ± 0.05 *b*	NS
ΣMUFA	42.82 ± 1.14 *a*	36.29 ± 1.21 *b*	68.11 ± 1.26 *c*	49.99 ± 4.19 *d*	42.91 ± 1.15 *a*	34.91 ± 0.43 *b*	63.40 ± 4.28 *c*	49.35 ± 4.35 *d*	NS
16:1n7	3.12 ± 0.18 *a*	5.23 ± 0.67 *b*	0.80 ± 0.12 *c*	1.87 ± 0.42 *c*	2.86 ± 0.33 *a*	6.20 ± 0.46 *b*	1.43 ± 0.41 *c*	2.42 ± 0.39 *ab*	NS
18:1n9c	33.09 ± 1.32 *a*	24.76 ± 1.59 *b*	63.73 ± 1.47 *c*	43.84 ± 4.64 *d*	32.65 ± 1.29 *a*	23.08 ± 0.54 *b*	62.64 ± 0.68 *c*	38.52 ± 0.83 *ab*	NS
18:1n9t	0.23 ± 0.02	0.34 ± 0.08	0.38 ± 0.07	0.23 ± 0.04	0.28 ± 0.03	0.46 ± 0.23	0.33 ± 0.07	0.41 ± 0.19	NS
18:1n7	5.99 ± 0.26 *a*	5.69 ± 0.23 *a*	2.91 ± 0.17 *b*	3.78 ± 0.31 *c*	6.86 ± 0.23 *a*	4.89 ± 0.20 *b*	3.03 ± 0.16 *c*	3.39 ± 0.18 *c*	NS
20:1n9	0.49 ± 0.02 *a*	0.45 ± 0.02 *a*	1.46 ± 0.08 *b*	0.92 ± 0.14 *c*	0.48 ± 0.04 *a*	0.37 ± 0.02 *a*	0.99 ± 0.10 *b*	0.77 ± 0.17 *c*	*
22:1n9	0.29 ± 0.13	0.21 ± 0.08	0.19 ± 0.01	0.20 ± 0.03	0.16 ± 0.03	0.24 ± 0.09	0.18 ± 0.02	0.20 ± 0.06	NS
ΣPUFA	16.30 ± 0.94 *ab*	17.47 ± 0.82 *a*	9.18 ± 0.44 *c*	15.02 ± 1.79 *b*	18.89 ± 0.78 *a*	18.96 ± 0.42 *a*	10.68 ± 1.24 *b*	14.84 ± 1.27 *b*	NS
16:3n4	0.34 ± 0.04 *ab*	0.23 ± 0.02 *ab*	0.16 ± 0.02 *b*	0.22 ± 0.03 *b*	0.37 ± 0.04 *a*	0.30 ± 0.05 *a*	0.19 ± 0.02 *b*	0.28 ± 0.03 *ab*	NS
18:2n6	2.52 ± 1.21 *ab*	1.36 ± 0.24 *a*	0.61 ± 0.04 *b*	0.82 ± 0.08 *b*	3.24 ± 0.98 *a*	1.45 ± 0.04 *b*	0.94 ± 0.04 *c*	1.11 ± 0.07 *c*	*
18:3n6	0.230 ± 0.005	0.234 ± 0.029	0.194 ± 0.006	0.226 ± 0.022	0.324 ± 0.016 *a*	0.265 ± 0.024 *ab*	0.201 ± 0.009 *c*	0.235 ± 0.011 *bc*	NS
20:2	1.48 ± 0.12 *ab*	3.03 ± 0.24 *b*	1.69 ± 0.11 *a*	3.23 ± 0.48 *b*	0.97 ± 0.15 *a*	2.00 ± 0.18 *b*	1.23 ± 0.27 *a*	1.83 ± 0.23 *b*	*
20:3n6	1.45 ± 0.07 *ab*	1.43 ± 0.08 *a*	0.72 ± 0.05 *b*	1.09 ± 0.12 *c*	1.66 ± 0.06 *a*	1.46 ± 0.05 *a*	0.76 ± 0.08 *b*	1.03 ± 0.08 *c*	NS
20:4n6	8.68 ± 0.39 *ab*	9.18 ± 0.50 *a*	5.08 ± 0.25 *b*	8.07 ± 0.94 *a*	11.12 ± 0.28 *a*	11.34 ± 0.38 *a*	6.52 ± 0.78 *b*	8.82 ± 0.85 *c*	*
20:4n3	0.15 ± 0.06	0.19 ± 0.10	0.07 ± 0.01	0.15 ± 0.04	0.14 ± 0.03	0.28 ± 0.11	0.08 ± 0.02	0.21 ± 0.09	NS
20:5n3	0.54 ± 0.14 *ac*	0.95 ± 0.14 *b*	0.16 ± 0.02 *a*	0.64 ± 0.15 *bc*	0.39 ± 0.05 *a*	0.92 ± 0.13 *b*	0.27 ± 0.06 *a*	0.63 ± 0.11 *ab*	NS
22:5n3	0.43 ± 0.05 *a*	0.52 ± 0.03 *b*	0.31 ± 0.01 *c*	0.39 ± 0.04 *ac*	0.53 ± 0.01 *a*	0.61 ± 0.02 *a*	0.33 ± 0.02 *b*	0.41 ± 0.04 *b*	NS

Data are mean ± SEM (*n* = 4–5/group). Different lower-case letters denote statistical difference in FA abundance between NEFA treatments within a differentiation state (CT or SCT) (*p* < 0.05). * Denotes a differentiation state-dependent difference in FA abundance between BeWo CT and SCT cells (*p* < 0.05); NS indicates no statistical significance between differentiation states.

**Table 2:** Neutral lipid profiles of NEFA-treated BeWo trophoblast cells.

Neutral Lipid Species	CT				SCT			
	BSA Ctrl	PA	OA	P/O	BSA Ctrl	PA	OA	P/O
Cholesterol Esters	10.07 ± 2.66	25.46 ± 9.52	11.29 ± 2.28	15.44 ± 4.39	9.03 ± 1.69	17.56 ± 6.19	12.19 ± 4.33	19.18 ± 7.54
Free Fatty Acids	10.46 ± 3.67	11.91 ± 5.12	8.90 ± 3.36	10.05 ± 4.04	11.33 ± 3.69	9.93 ± 2.81	7.81 ± 2.89	8.72 ± 2.55
Triglycerides	15.99 ± 5.14 *a*	17.12 ± 2.63 *a*	39.47 ± 2.59 *b*	18.35 ± 2.88 *a*	14.34 ± 1.97 *a*	14.74 ± 1.51 *a*	30.86 ± 4.95 *b*	17.96 ± 1.19 *a*
Free Cholesterol	61.80 ± 6.21 *a*	43.6 ± 5.91 *bc*	37.84 ± 1.32 *b*	53.79 ± 3.88 *ac*	62.88 ± 3.19 *a*	56.39 ± 5.57 *ab*	46.40 ± 4.62 *b*	52.24 ± 6.66 *ab*
Diacylglycerols	1.68 ± 0.32	1.91 ± 0.77	2.51 ± 0.35	2.37 ± 0.42	2.43 ± 0.89	1.38 ± 0.24	2.75 ± 0.07	1.90 ± 0.26

Data are percent of neutral lipid fraction (mean ± SEM; *n* = 5/group). Different lower-case letters denote statistical differences between NEFA-treatments within each differentiation state (CT or SCT cells), *p* < 0.05.

## Data Availability

Microarray data are available in the National Center for Biotechnology Information (NCBI) Gene Expression Omnibus (GEO) database (GSE197385). A full list of metabolome peak pairs and their high-confidence/putative identifications are available in Supplementary Table S7. A full list of lipidome peak intensities and their high-confidence/putative identifications are available in Supplementary Table S10.

## Supplementary Materials

The following supporting information can be downloaded at: https://www.mdpi.com/article/10.3390/metabo13080883/s1, Figure S1: Multivariate visualization of the degree of separation between metabolite profiles in NEFA-treated BeWo CT cells. (A) Unsupervised principal component analysis (PCA) and (B) supervised partial least squares discriminant analysis (PLS-DA) plots were constructed to visualize the degree of difference in metabolite profiles between PA, OA, P/O and BSA-control cultures; Figure S2: Multivariate visualization of the degree of separation between lipidome profiles in NEFA-treated BeWo CT cells. (A) Unsupervised principal component analysis (PCA) and (B) supervised partial least squares discriminant analysis (PLS-DA) plots were utilized to visualize the degree of difference in lipidome profiles between PA, OA, P/O and BSA-control cultures; Table S1: Forward and reverse primer sequences used for quantitative Real-Time PCR validation of NEFA treatment microarrays; Table S2: Summary of differentially expressed genes (DEGs) identified in PA, OA and P/O-treated BeWo CT cells compared to BSA-control cultures; Table S3: Summary of shared and unique Differentially Expressed Genes (DEGs) in NEFA-treated BeWo CT cells; Table S4: Summary of over-representation enrichment analysis of NEFA treated BeWo CT transcriptome profiles highlighting the top 10 pathways (by FDR corrected p-value) in the Wikipathway, Reactome and KEGG functioanl datasets, as well as the Gene Ontology (GO) biological processes and molecular function datasets; Table S5: Summary of Gene Set Enrichment Analysis of NEFA treated BeWo CT transcriptome profiles showing top 10 upregulated and downregulated pathways (by FDR corrected p-value) in the Wikipathway, Reactome and KEGG functional databases, as well as the Gene Ontology (GO) molecular function and biological pro-cesses datasets; Table S6: Ranked gene lists (signal-to-noise ratio) of NEFA-treated BeWo CT cells; Table S7: Summary of identified metabolites, and metabolite peak-pair data for NEFA-treated BeWo CT cells; Table S8: Summary of differentially abundant metabolites in NEFA-treated BeWo CT cells; Table S9: Summary of differenitally abundant metabolites shared between 2 or more NEFA-treatment groups; Table S10: Summary of identified lipid species, and respective peak in-tensity data for NEFA-treated BeWo CT cells; Table S11: Summary of abbreviations of lipid sub-classes identified in untargeted lipidomic analysis; Table S12: Summary of differentially abundant lipid species in NEFA-treated BeWo CT cells; Table S13: Summary of differenitally abundant lipid species (DALs) shared between 2 or more NEFA-treatment groups, and unique to each NE-FA-treatment group.
