# Supplementary material for: An Integrated Multi-OMICS Approach Highlights Elevated Non-Esterified Fatty Acids Impact BeWo Trophoblast Metabolism and Lipid Processing
Source: Metabolites. 2023 Jul 25;13(8):883. doi: 10.3390/metabo13080883 (PMC10456680; doi:10.3390/metabo13080883)
Supplement: Supplementary file 1 [file metabolites-13-00883-s001.zip › S2 file - metabolites-2476885-Supplementary figures.pdf]

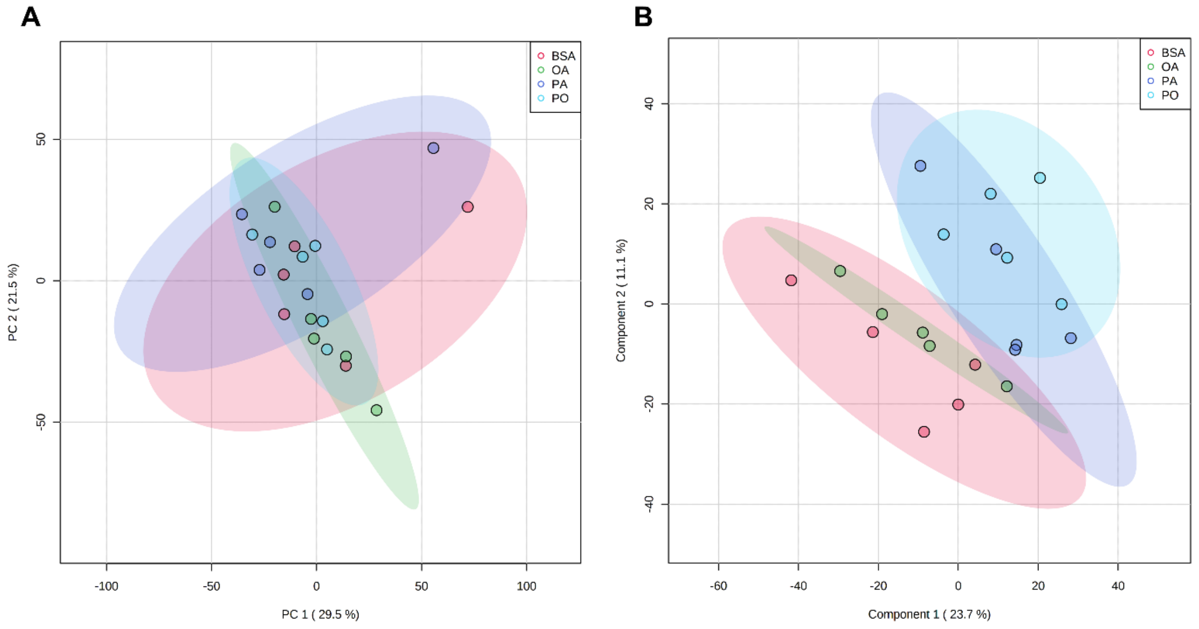

Supplementary Figure S1. Multivariate visualization of the degree of separation between metabolite profiles in NEFA-treated BeWo CT cells. (A) Unsupervised principal component analysis (PCA) and **(B)** supervised partial least squares discriminant analysis (PLS-DA) plots were constructed to visualize the degree of difference in metabolite profiles between PA, OA, P/O and BSA-control cultures.

up

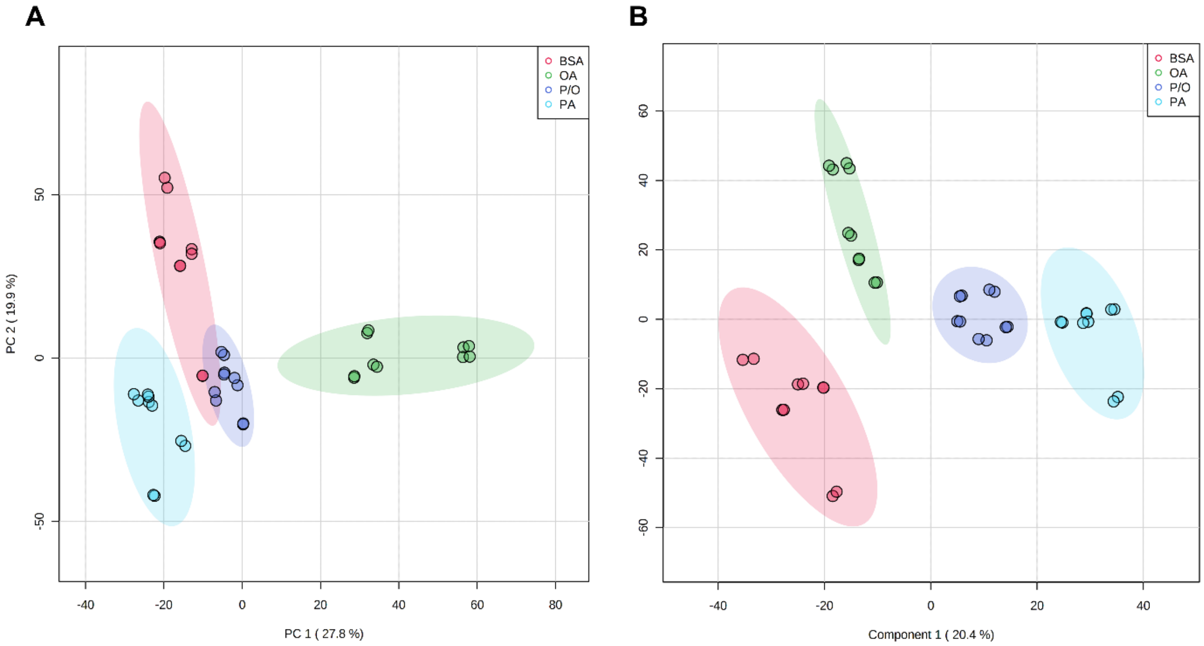

Supplementary Figure S2. Multivariate visualization of the degree of separation between lipidome profiles in NEFA-treated BeWo CT cells. (A) Unsupervised principal component analysis (PCA) and **(B)** supervised partial least squares discriminant analysis (PLS-DA) plots were utilized to visualize the degree of difference in lipidome profiles between PA, OA, P/O and BSA-control cultures.
